# *Mycobacterium tuberculosis* antigen-containing exosomes reinforce BCG vaccine efficacy by augmenting long-term protection and memory response against experimental tuberculosis in BALB-C mice

**DOI:** 10.3389/fimmu.2026.1742207

**Published:** 2026-03-13

**Authors:** Manu Sharma, Sher Afghan, Amit Singh, Iqbal Alam, Meetu Agarwal, Mohd Shahid, Shaikh Muhammad Atif, Saif Khan, Syed Saima Malik, Vengala Rao Yenuganti, Mairaj Ahmed Ansari

**Affiliations:** 1 Department of Biotechnology, School of Chemical and Life Sciences, Jamia Hamdard, New Delhi, India; 2 Department of Physiology, Hamdard Institute of Medical Sciences and Research (HIMSR), Jamia Hamdard, New Delhi, India; 3 National JALMA Institute of Leprosy and Other Mycobacterial Diseases, Agra, Uttar Pradesh, India; 4 Department of Molecular Medicine, School of Interdisciplinary Sciences and Technology, Jamia Hamdard, New Delhi, India; 5 College of Pharmacy & School of Graduate and Post Doctoral Studies, Rosalind Franklin University of Medicine and Science, North Chicago, IL, United States; 6 Department of Medicine Infectious Diseases, University of Colorado Anschutz Medical, Aurora, CO, United States; 7 Department of Biological Sciences, SRM University-AP, Amaravati, Andhra Pradesh, India

**Keywords:** BCG vaccine booster, exosomes as candidate vaccine, long term protection, T cell antigen bearing exosomes, T cell memory responses, tuberculosis vaccine, exosome as booster

## Abstract

Tuberculosis (TB), caused by *Mycobacterium tuberculosis* (*M.tb.*), affects one-third of humanity. Despite the availability of effective drug regimens, complete eradication of *M.tb.* remains challenging due to prolonged treatment duration. Additionally, MDR-TB and co-infection with HIV further exacerbate disease severity. The Bacille Calmette–Guérin (BCG) vaccine has shown inconsistent efficacy due to the absence of Th-1 antigens. Hence, there is a critical need for either a novel vaccine candidate or an efficient booster to enhance BCG’s prophylactic efficacy. In this study, in-house prepared *M.tb.*-infected alveolar macrophage-derived exosomes (Rv-Exo) and ESAT-6-containing exosomes (ESAT-6 Exo) were characterized based on size, purity, and pathogen-associated molecular patterns (PAMPs), and their epitope mapping was also performed. These *M.tb.* protein-containing exosomes (MPEs) were utilized for immunization, either alone or as a booster to BCG, and evaluated in BALB/c mice against experimental *M.tb.* challenge. Our results demonstrate that ESAT-6 Exo and Rv-Exo, either alone or as a BCG booster, enhanced Th-1-biased immune responses by activating CD4^+^ and CD8^+^ T cells, increasing memory T-cell populations, and significantly reducing the *M.tb.* burden in the lungs, spleen, and lymph nodes of infected mice. These findings highlight the potential of MPE as a promising strategy against TB especially in the BCG-vaccinated population.

## Graphical Abstract

**Figure:**
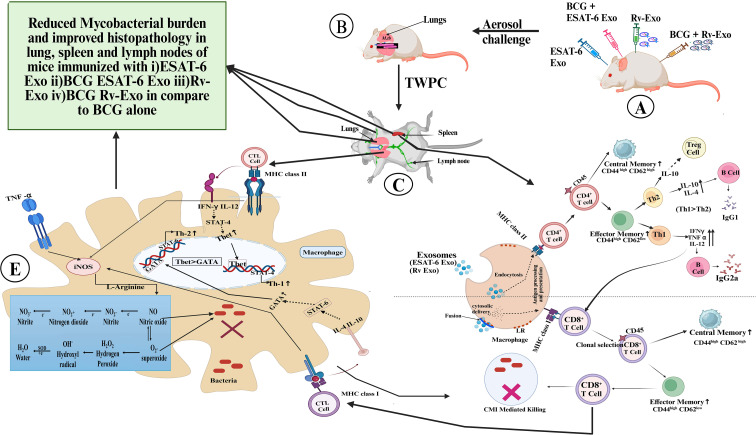
The M.tb. antigen bearing exosomes (Rv-Exo and ESAT-6 Exo) deliver antigen by fusion or endocytosis to boost BCG efficacy. They induce functional T-cells population, Th-1 skewed immuneresponse, effector/central CD4/CD8 T-cells memory, elevate iNOS/Nitric oxide, and reduce bacterial-burden in lungs, spleen and lymph nodes at twelve-weeks post-challenge (TWPC) in murine model.

## Introduction

*Mycobacterium tuberculosis* (*M.tb.*) is an intracellular bacterium that causes tuberculosis (TB), a major public health concern, affecting one-third of the global population (1). The pathogen is transmitted via inhalation of aerosolized droplets from infected individuals. Although 90% of infected individuals remain asymptomatic (latent TB), approximately 10% develop active disease^i^. As per the 2025 global TB report, in 2024, TB affected an estimated 10.7 million people worldwide, with a higher prevalence in immunocompromised individuals, particularly those with HIV, which depletes CD4^+^ T cells and weakens immune defenses against TB pathogens and increases reactivation risk (2). The World Health Organization (WHO) reported that TB caused 1.25 million deaths in 2024, including 150,000 among people with HIV, whereas 82% of patients with TB had documented HIV testing (2). Over recent years, global TB incidence has shown an uneven decline, but new diagnoses rose to 8.2 million in 2023 from 7.5 million in 2022 (2).

Current diagnostic tests for TB have significant limitations despite their ability to detect infection. For instance, skin tests yield false positives in Bacille Calmette–Guérin (BCG)-vaccinated individuals, while blood tests require specialized facilities (3). Sputum microscopy has low sensitivity, and cultures take weeks (4). Additionally, TB treatment is prolonged (6–9 months), often complicated by adherence challenges and side effects (5). Drug-resistant TB requires longer, more toxic regimens, while latent TB infection necessitates months of preventive therapy (6). Despite advances in diagnostics and therapeutics, TB remains a significant global health burden, underscoring the urgent need for an effective vaccine.

Vaccines offer a promising strategy for controlling this devastating pathogen. The BCG vaccine, derived from *Mycobacterium bovis*, is currently the only licensed vaccine for the prevention of TB in humans (7). However, the protective efficacy of BCG against adult pulmonary TB remains variable and controversial, largely due to the absence of key virulence factors containing T-cell epitopes. Notably, attenuation of BCG resulted from deletion of the RD1 locus (9.5 kb), which encompasses the *Rv3871–Rv3879c* gene cluster encoding 14 open reading frames. This deletion eliminates the expression of important *M.tb.* antigens, including the culture filtrate protein (CFP-10) and early secreted antigenic target (ESAT-6), both of which are secreted via the ESX-1 secretion system and are recognized as essential virulence determinants, thereby contributing to the reduced immunogenicity of BCG.

These limitations underscore the urgent need for novel vaccine candidates or booster strategies that could enhance BCG efficacy by complementing T-cell epitopes. As of 2022, according to WHO, 16 TB vaccine candidates were under clinical trial; of these, 6 had advanced to phase III trials, including M72/AS01E, a sub-unit vaccine, and VPM002, a recombinant BCG vaccine (German firm MTB) (8), both of which are considered among the most promising candidates. Additional vaccine candidates are in the earlier stages of clinical development, including live-attenuated strains, protein subunit vaccines, and viral vector-based approaches (9).

Conventional protein-based or killed whole-cell microbes vaccines predominantly elicit antibody responses (humoral) but offer limited T helper (Th) cell activation (10). Antibody-mediated immunity alone is ineffective for protection against intracellular pathogens such as *M.tb.*, which requires robust pathogen-specific CD8^+^ cytotoxic and CD4^+^ helper T cell-mediated immunity for effective clearance (11). Notably, *M.tb.* evades host T cell-mediated immunity by downregulating major histocompatibility complex (MHC) class II expression on macrophages, thereby impairing antigen presentation and T-cell activation (12). Antigen dose and duration of exposure critically influence T-cell memory differentiation. Effector memory T cells (TEM) provide immediate protection during primary infection but decline rapidly, whereas central memory T cells (TCM) persist long-term and mediate rapid response upon reinfection. Thus, establishing durable T-cell immune memory is a key determinant of successful TB vaccination.

Pathogen-specific CD4^+^ T cells regulate immune responses through the secretion of Th-1 [interferon-gamma (IFN-γ), IL-2, and TNF-α], and Th-2 (IL-10 and IL-4) cytokines. During *M.tb.* infection, Th-1 cytokine response is often suppressed; in contrast, an effective vaccine must induce a Th-1-biased immune response to promote bacterial clearance. These specific responses are regulated by transcription factors Tbx-21 (Th-1) and GATA (Th-2) signaling (13). Elevated levels of reactive oxygen species (ROS) and reactive nitrogen species (RNS) further corroborate enhanced pathogen-specific immune activation with reduced bacterial burden in infected tissues (14).

Exosomes are small extracellular vesicles (EVs) (30–150 nm) secreted by most eukaryotic cells and play a vital role in intercellular communication by transporting proteins, lipids, and nucleic acids. Once considered cellular waste products, exosomes are now recognized for their potential in diagnostics and therapeutics, particularly in infectious diseases (15). Exosomes derived from *M.tb.*-infected cells contain mycobacterial antigens and virulence factors capable of modulating host immune responses, making them promising candidates for vaccine delivery (16, 17). Their lipid bilayer structure preserves antigen stability, facilitates efficient antigen presentation, and promotes robust immune responses with a lower risk of adverse effects compared with conventional adjuvants (17, 18). Furthermore, exosomes can be engineered to carry single or multiple antigens, allowing for the development of multivalent vaccines suited to complex diseases requiring broad and coordinated immune responses (19). Current TB vaccine strategies include modifying BCG to enhance its immunogenicity (e.g., VPM-1002 and rBCG30) as well as a prime-boost approach in which BCG vaccination is followed by subunit or viral vector-based boosters (20). We have previously demonstrated that archaeosome-encapsulated ESAT-6 provoked enhanced Th-1-biased immune responses and CTL activation in murine models (20).

In the present study, we systematically evaluated the vaccine efficacy of in-house-prepared exosome-based vaccine candidates, Rv-Exo and ESAT-6 Exo, against TB, both as stand-alone vaccine formulation and within a “prime-boost” vaccination strategy. Our findings demonstrate that exosomes bearing *M.tb.* antigens significantly enhance antigen presentation and modulate immune responses, resulting in elevated *M.tb*-specific Th-1-biased and CTL-mediated immunity. The ESAT-6, a key virulence factor of *M.tb.* and a potent inducer of cell-mediated immunity, was particularly effective when delivered via exosomes derived from H37Rv-infected alveolar macrophages, enabling precise immune regulation and robust CTL activation. When the BCG-immunized animals were boosted with ESAT-6 Exo or Rv-Exo, we observed (i) increased Th-1-biased cytokine levels in serum; (ii) elevated population of IFN-γ-, TNF-α-, and IL-2-producing Th-1 cells; (iii) a higher IgG2a:IgG1 ratio; (iv) dose-dependent enhancement of *M.tb*-specific lymphocyte proliferation; (v) upregulated T-bet expression; (vi) significantly reduced bacterial burden in vital organs; and (vii) diminished bacterial presence in tissue samples. Collectively, these results support the potential of exosome-based vaccines containing ESAT-6 or derived from H37Rv-infected alveolar macrophages as a novel and effective strategy for TB vaccination. The promising efficacy observed in both naïve and booster settings underscore the need for further preclinical optimization and clinical evaluation to assess the long-term protective immunity.

## Material and methods

### Chemicals and reagents

Unless otherwise mentioned, all standard reagents were purchased from Sigma-Aldrich Merck (MA, USA). The following reagents were procured from BD Biosciences: Middle-brook 7H9 broth (271310); Middle-brook 7H11 medium (212203); and oleic acid, albumin, dextrose, and catalase (OADC). Cell culture media (RPMI-1640), fetal bovine serum, and antibiotic and antimycotic solution were purchased from Gibco, Thermo Fisher Scientific, and plasticwares were purchased from corning Falcon.

### ESAT-6 protein expression and purification

The pET expression vectors Pmrlb.7 were procured from BEI resources. The Plasmid Miniprep kit was purchased from Thermo Scientific (USA), and the Gel extraction kit used for plasmid preparations and DNA purification processes was from Qiagen. To concentrate expressed protein, Amicon-Ultra was used (molecular mass cutoff, 3.5 kDa; Millipore, Bangalore, India). The ESAT-6 was purified over a nickel/nitrilotriacetic acid (Ni/NTA) matrix (Qiagen) using a standard protocol under denaturing conditions, as per the manufacturer’s instructions, The eluted fractions were checked for purity by SDS/PAGE (15% gel) as well as Western blot analysis following the standard method. The protein was refolded by dialyzing it against refolding buffer containing 25 mM NaH_2_PO_4_, 100 mM NaCl, 1 mM 20 mM NaH_2_PO_4_, 50 mM NaCl, and 0.1% NaN_3_, pH 6.5.

### Bacteria and alveolar macrophage cell culture

*M. tuberculosis* H37Rv strains were kindly provided by the ICMR National JALMA Institute for Leprosy and other Mycobacterial disease, Agra, India. It was cultured into Middle-brook 7H9 broth containing 0.2% glycerol and 0.05% Tween-80 supplemented with albumin, dextrose, and catalase. The viability of the bacteria was determined by cultivating them on Middle-brook 7H11 medium supplemented with OADC (BD Biosciences, New Jersey, USA) and counting the colony-forming units (CFUs). The mouse alveolar macrophage cell line was purchased from ATCC USA. MH-S cells (ATCC No. CRL-2019) were cultured in wells or flasks with RPMI-1640 containing 10% exosome-depleted FBS, 0.05 mM β-mercaptoethanol, 100 U/mL penicillin, and 0.1 mg/mL streptomycin at 37 °C with 5% CO_2_.

### Alveolar macrophages cells infection with *M.tb.* H37Rv

Mouse alveolar macrophage (MH-S) cell lines were starved for 24 h and then infected with 5 multiplicity of infection (MOI) of H37Rv for 24 h. The infected macrophages were then washed three times with PBS. The cell culture supernatant was collected and centrifuged at 10,000 rpm at 4 °C for 10 min to remove any bacilli. In addition, the culture supernatant was filtered with a 0.22-µm filter.

### Preparation of *M.tb.* antigen-bearing exosomes

Mouse alveolar macrophages (70%–80% confluence) were cultured in RPMI-1640 supplemented with 10% exosome-depleted FBS at 37 °C and 5% CO_2_ and either left uninfected (UI) or infected with *M.tb.* H37Rv at an MOI of 5. Culture supernatants were collected, centrifuged at 10,000 rpm for 10 min at 4 °C, filtered through 0.22-µm filters, and ultracentrifuged at 100,000 *g* for 2 h at 4 °C (Beckman Coulter, USA) to pellet exosomes (Rv-Exo). Pellets were resuspended in 150 mM saline, quantified by BCA assay, and stored at −80 °C.

ESAT-6-loaded exosomes were prepared from UI macrophage-derived exosomes by sonication. Purified exosomes were resuspended in 150 mM saline and mixed with purified ESAT-6 (400 µg/200 µL). The mixture was sonicated for 5 min at 4 °C using a bath sonicator (20 kHz; 30 s pulse cycles), followed by multiple freeze–thaw cycles to enhance encapsulation. ESAT-6-loaded exosomes were pelleted by centrifugation at 17,000 rpm for 2 h at 4 °C, and unencapsulated ESAT-6 was collected from the supernatant. Exosomes were lysed with 1× RIPA buffer, protein was estimated by BCA assay, and then encapsulation efficiency was determined by ESAT-6 immunoblotting and densitometry analysis of the concerned bands.

### Electron microscopy

Transmission electron microscopy (TEM; Talos L 120C, Thermo Scientific, USA) was used to study the morphology of H37Rv-infected alveolar macrophage cell-derived exosomes. After being carefully positioned on a carbon-coated 300-mesh copper grid for 20 min, purified exosome preparations (25 μL) were diluted with an equal volume of 4% paraformaldehyde at 4 °C for 30 min. They were subsequently fixed for 5 min using 1% glutaraldehyde. The grids were cleaned twice, contrasted with 2% uranyl acetate, and then cleaned twice more. The software was used to examine the exosome pictures from TEM in order to determine the exosome radius.

### NTA analysis of exosomes

All samples were diluted 1:1,000 (V:V) in PBS. The prediction of ideal measurement concentrations was determined by pre-testing the ideal particle per frame value (74 particles/frame). The software provided by the manufacturer was used as default settings for EVs. For each measurement, one cycle was performed by scanning 11 cell positions each and capturing frames per position (video setting: high) under the following settings: focus: autofocus; camera sensitivity for all samples: 92.0; shutter: 200; and cell temperature: 25 °C. After capture, the videos were analyzed by the in-built ZetaView Software 8.05.16 SP3 with specific analysis parameters: maximum particle size: 1,000, minimum particle size 10, and minimum particle brightness: 30 (21). The data were analyzed for particle size as well as number and presented here in the form of a bar graph.

### Western blot analysis

Antibodies against NF‐κB (AF5006, 1:1,000), COX‐2 (AF7003, 1:1,000), and inducible nitric oxide synthase (iNOS) (AF0199, 1:1,000) were bought from Affinity Biosciences (USA). Antibodies against CD63 mouse monoclonal antibody (SC-5275, 1:500) and Calnexin mouse monoclonal antibody (SC-23954, 1:1,000) were procured from Santa Cruz Biotechnology (USA). pIRF3 (4947, 1:1,000), pSTING (85735, 1:1,000), tubulin (2144, 1:5,000), HRP-conjugated anti-rabbit IgG (7074, 1:5,000), and anti-mouse IgG (7076, 1:5,000) antibodies were from Cell Signaling Technology (USA). Precision Plus Protein Standards and 161-0373 Protein markers were purchased from Bio-Rad (USA).

Exosomes were lysed by radioimmunoprecipitation assay (RIPA) buffer (20-188, Millipore-Sigma) supplemented with protease and phosphatase inhibitor cocktail (Ab-271306, Abcam). An equal amount of exosomal protein was subjected to electrophoresis using 12% polyacrylamide gel (Bio-Rad, USA) and transferred to PVDF membranes (Millipore, Bedford, MA, USA). Membranes were blocked with TBST containing 5% skimmed milk and 0.1% Tween-20, then incubated with primary antibody, followed by HRP-labeled secondary antibody. The blots were developed using ECL-specific reagents (Bio-Rad, USA). The images were captured using Chemidoc MP and analyzed using the CFX Maestro Software version 2.2 (Bio-Rad, USA).

### Exosome protein delivery assay

For protein labeling of EVs, containing 200–500 μg of protein, they were resuspended in 500 μL of PBS. A 500× labeling dye (Exoglow protein-Protein EV labeling Kit, EXOGP400A-1, System Biosciences) was then added to the EV preparation at a 1:500 dilution, and the mixture was incubated at 37 °C with shaking (350 rpm) for 20 min. Subsequently, 167 μL of ExoQuick-TC was added to the solution, and the mixture was incubated overnight at 4 °C. The EV–dye complex was then centrifuged at 10,000 rpm for 10 min, and the supernatant was carefully aspirated from the corner of the tube. The labeled EV pellet was resuspended in 300 μL of PBS. J-774 macrophages (NCCS, Pune, India) were seeded into an 8-well chamber slide and incubated with labeled exosomes for 10 min and 1 h at 37 °C under 5% CO_2_ to evaluate uptake kinetics. Post-incubation, unbound exosomes were removed by PBS washing, and cells were fixed with 4% paraformaldehyde (PFA) for 20 min at room temperature. DAPI was used to stain the nucleus (62248, Thermo Scientific, USA). Images were taken using Evos-M7000 (Invitrogen, Thermo Scientific, USA).

### Mass spectrometry of H37Rv-infected cell-derived exosomes

LC-MS experiments were outsourced and performed on a Morpheus (Agilent, revision 272) that was linked to a Thermo QE Plus. Protein (1 μg) from each sample was inoculated into a 50-cm-long, 3.0-mm-thick C18 column (Thermo Fisher Scientific). At a flow rate of 300 nL/min, a 0%–40% gradient of buffer B (80% acetonitrile, 0.1% formic acid) was used to elute the peptides, which were then sent to the MS analyzer. For 60 min, LC gradients were run. At a resolution of 70k, MS1 spectra were taken with the Orbitrap. Dynamic exclusion was used for 10 s, and all charge states for sequences given the precursor were taken out of the equation. MS2 spectra were taken at a resolution of 17,500.

### Epitope prediction of T cells and B cells

Raw data obtained from proteome analysis were used to generate the UniProt ID using the NCBI database, and at the end, we had FASTA sequence of all the protein present in exosomes in FASTA format. Further, the selected proteins were subjected to T- or B- cell specific epitope prediction using server NetMHCpan El 4 version of Immune Epitope Databases (IEDB) software. The IEDB tool uses validated benchmarking methods to predict MHC molecular binding, antigen processing, TCR recognition, and B-cell epitopes. MHC class I-restricted CD8^+^ cytotoxic T lymphocyte (CTL) epitope selected sequences compatible to respective common human leukocyte antigen (HLA) alleles (i.e., H2-db and H2-kb) having an epitope length of 9 amino acids. Similarly, we identified MHC class II-restricted CD4^+^ helper T lymphocyte (HTL) epitopes of several common HLA alleles (e.g., H2-IAb) using the IEDB-recommended 2.22 prediction method with 15 epitopes. The BepiPred 2.0 server predicted linear B-cell epitopes of selected protein sequences. In the next step, the predicted value is increased sequentially and considered more likely than threshold (default 0.5) epitopes.

### Mice maintenance and experimentation

All animal experiments were approved by the National JALMA Animal Ethical Committee and the Jamia Hamdard Institutional Ethics Committee (Approval No. 1566/2019) and conducted in accordance with CPCSEA guidelines. Female BALB/c mice (6–8 weeks old) were maintained under specific pathogen-free conditions at Jamia Hamdard and transferred to the animal biosafety level 3 (ABSL-3) facility at the ICMR-National JALMA Institute of Leprosy and Other Mycobacterial Diseases, Agra, India, where they were acclimatized for 10 days prior to experimentation.

### Immunization and challenge with mycobacterial infection

Mice were immunized subcutaneously with free ESAT-6 antigen, ESAT-6-encapsulated exosomes (ESAT-6 Exo), Rv-Exo, BCG alone, or BCG followed by boosting with ESAT-6 Exo or Rv-Exo. PBS and sham exosomes served as controls. Antigens were administered at 100 µg per mouse in a total volume of 100 µL, and exosome formulations were delivered at 1,000 µg per injection. BCG-immunized groups received a single intradermal dose of *M. bovis* BCG (Danish strain, 1×10^6^ CFU/mouse). Booster immunizations were administered on day 15 using the same route. Two weeks post-booster, mice were challenged with aerosolized *M. tuberculosis* H37Rv using a Glas-Col aerosol exposure system (IN, USA) delivering a bacterial suspension of 1×10^7^ CFU/mL. Infected animals were housed in IVEC chambers for the duration of the study. Bacterial deposition in lungs was quantified 24 h post-challenge. At 6 and 12 weeks post-challenge, lungs, spleens, and lymph nodes (*n* = 3/group/time point) were harvested for bacterial burden analysis. Organs were homogenized in Middlebrook 7H9 medium (Polytron PT 3100, Indonesia), serially diluted, and plated on 7H11 agar supplemented with OADC. Thiophene-2-carboxylic acid hydrazide (TCH, 2 mg/mL, Sigma-Aldrich, T25001) was included to inhibit BCG growth where appropriate. Plates were incubated at 37 °C for 3–4 weeks, and CFUs were calculated per gram of tissue.

### Serum cytokines and antibody isotype analysis

Serum samples collected pre-immunization, post-boost, and 12 weeks post-challenge were analyzed for IgG1 (KLM1629), IgG2a (KLM1488), IFN-γ (KB2011), IL-12 (KLM0020), IL-4 (KLM0051), and IL-10 (KB2072) using GENLISA sandwich ELISA kits (Krishgen BioSystems, USA, catalog numbers as specified). Assays were performed according to the manufacturer’s instructions, and absorbance was measured at 450 nm using a Synergy H1 multimode reader (BioTek, USA).

### Lymphocyte isolation, proliferation, and flow cytometry

Mice belonging to various immunized groups were sacrificed at the 6- and 12-week time points, both after vaccination and post infection. Splenocytes were isolated from vaccinated and infected mice at 6 and 12 weeks, processed into single-cell suspensions, and cultured in RPMI 1640 supplemented with 10% FBS. For immunophenotyping, cells were stimulated with purified protein derivative (PPD, 5 µg/mL) or ESAT-6 (10 µg/mL) and analyzed by flow cytometry following surface and intracellular staining. Fluorochrome-conjugated antibodies against CD3ϵ APC (clone 145 2C11, 561826), CD4 BV786 (clone GK1.5, Cat. No. 563331), CD8 APC-H7 (clone 53 6.7, Cat. No. 560247), CD44 BV480 (clone IM7, Cat. No. 566200), CD45 BB700 (clone 30 F11, Cat. No. 566440), CD62L BB515 (clone MEL14, Cat. No. 565261), IL-2 PE (clone JES6-5h4, Cat. No. 554428), TNF-α PECy7 (clone MP6 XT22, 561041), IL-4 BV711 (clone 11B11, Cat. No. 564005), and IL-10 BV605 (clone JES5 16E3, Cat. No. 564082) were obtained from BD Biosciences (USA). One microliter of 1:100 brefeldin (555029, BD Biosciences) was used for intracellular cytokine accumulation. After washing, Cytofix/Cytoperm buffer (554714, BD Biosciences) was added for fixation and permeabilized before being stained with intracellular IL-2, TNF, IL-4, and IL-10 antibodies. Data were acquired on a BD FACS Lyric flow cytometer and analyzed using BD FACSuite software.

Antigen-specific lymphocyte proliferation was assessed using the MTT assay following stimulation with graded concentrations of ESAT-6 (1–100 µg/well) or PPD (5–20 µg/well) in a 96-well plate (1 million cells per well) in 200 µL of RPMI 1640 medium supplemented with 10% FBS. For the MTT reduction reaction, 10 μL of a 5 mg/mL MTT stock solution was added to each well, and the mixtures were incubated at 37 °C in the dark for 72 h. To dissolve the formazan crystals, 100 μL/well of a solubilizing buffer was added. The absorbance of the formazan products was determined by measuring the absorbance at 570 nm using a microplate reader (22).

### RNA isolation and qRT-PCR

At 12 weeks post-challenge, lungs, spleens, and lymph nodes were harvested from mice and homogenized in TRIzol reagent (Invitrogen, USA). Total RNA was isolated, assessed for integrity, and reverse-transcribed using the PrimeScript™ First Strand cDNA Synthesis Kit (Takara, Japan). Quantitative RT-PCR was performed using iQ SYBR Green Supermix (Bio-Rad, USA) to assess expression of TBX21, GATA-3, and IL1-β, normalized to GAPDH. Primer sequences are listed in **Table 1**.

**Table 1:** Primer sequence for various genes.

Primer name	Tm	GC %	Sequence
IL-1 β	F 59.35 R 57.30	55 50	GCCCATCCTCTGTGACTCAT AGGCCACAGGTATTTTGTCG
GAPDH	F 61.40 R 61.40	60 60	CTCCCACTCTTCCACCTTCG GCCTCTCTTGCTCAGTGTCC
Tbx-21	F 55.2 R 55.2	40 42.8	ACGTCTTTACTTTCCAAGAG GTACATGGACTCAAAGTTCTC
GATA-3	F 60 R 60	50 59.09	CCTCTGGAGGAGGAACGCTAAT GTTTCGGGTCTGGATGCCTTCT

### Histopathology and immunohistochemistry

Formalin-fixed lungs, spleens, and lymph nodes were paraffin-embedded, sectioned, and stained using Ziehl-Neelsen staining to visualize mycobacteria using a fluorescence microscope Evos M7000 Imaging system (Invitrogen, Thermo Scientific, USA). For immunohistochemistry, 4-μm-thick sections were probed with antibodies against IL-10 and IFN-γ for 12 h followed by HRP-conjugated secondary antibodies for 90 min and visualization with 0.05% diaminobenzidine (DAB) and 0.03% H_2_O_2_. After counterstaining with hematoxylin, sections were examined using the EVOS M7000 imaging system (Invitrogen, Thermo Fisher Scientific, USA).

### Nitric oxide assay

Serum nitric oxide (NO) levels were quantified using a Griess reagent assay kit (Sigma-Aldrich, G4410) in a 96-well plate according to the manufacturer’s instructions. Absorbance was measured at 540 nm, and NO concentrations were calculated using a sodium nitrite standard curve [Δ OD_{Std}_ = OD_{Std}_ − OD_{Blank}_], [Δ OD_{Sample}_ = OD_{Sample}_ − OD_{Blank}_]. Subsequently, the Δ OD Sample value was substituted into the equation to obtain the *x* value (in μM), representing the NO content.

### Statistical analysis

Data are expressed as mean ± standard error of mean. Differences between two groups or two treatments were compared using the Student’s two-tailed *t* test. Differences in three groups or more were compared using the one-way ANOVA (Tukey method). All data were analyzed using GraphPad Prism version 10.0 software. *p*-values < 0.05 (*), 0.01 (**), 0.001 (***), and 0.0001 (****) were considered statistically significant.

## Results

### Characterization of *M.tb.*-infected alveolar macrophage-derived (Rv-Exo) and ESAT-6-entrapped exosomes (ESAT-6 Exo)

**Figure 1:**
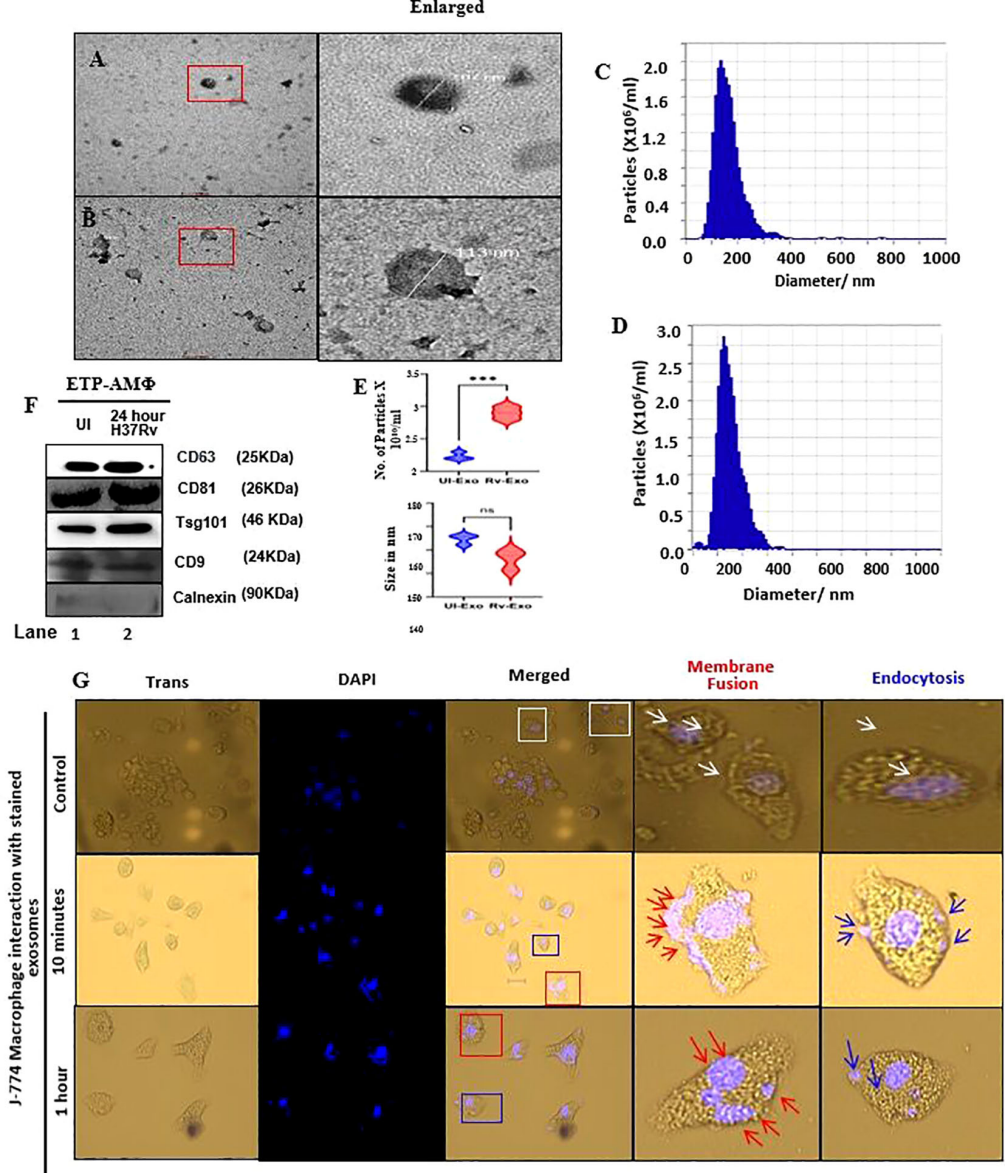
Isolation and characterization of exosomes for their size, origin, purity, and delivery method. The exosomes were isolated from murine alveolar macrophage either left uninfected (UI-Exo) or infected with 5 MOI of *M.tb.* H37Rv (Rv-Exo) by ultracentrifugation and were subjected to size depiction by TEM **(A)** UI-Exo and **(B)** Rv-Exo. The NTA-ZetaView analysis of UI-Exo **(C)** and Rv-Exo **(D)** depicts particle size distribution that is presented as a bar diagram (E, upper panel); the number of exosomes was also enumerated using NTA (**E**, lower panel). The lysates of UI-Exo and Rv-Exo were prepared using RIPA buffer, and 30 µg of protein from each group was subjected to Western blot analysis for CD-63, CD81, Tsg101, CD9, and Calnexin **(F)**. Exosomes isolated from J774 macrophages were labeled with Exo-Glow protein Blue overnight at 4 °C. The labeled exosomes were incubated with J774 macrophages for 10 min and 1 h in a CO_2_ incubator. Fusion of stained exosomes with the cell membrane was observed, indicating cargo release into the cytoplasm (**G**, Neither fusion nor endocytosis, white arrows; membrane fusion panel, red arrows). Endocytosis was also seen on the membrane as well as within the cytoplasm at 10 min and 1 h post-incubation, suggesting endosomal delivery of exosome content (**G**, Endocytosis panel, blue arrows). The white arrows depict nucleus stained by DAPI (top panel).

Exosomes isolated either from uninfected or *M.tb. H37Rv-*infected alveolar macrophages were subjected to electron microscopy (TEM). The TEM results suggest that the isolated exosomes vary in size ranging from 102 to 113 nm (**Figures 1A, B**). When particles were analyzed using NTA (zeta view), both UI-Exo (1C) and Rv-Exo (1D) showed 142.53 and 137.56 nm average size and number of particles were found to be 2.2 × 10^6^/mL and 2.86 × 10^6^/mL, respectively (**Figure 1E**). Interestingly, the number of particles significantly increased in the *M.tb.*-infected cells, which signifies the impact of infection in EV production (**Figure 1E**). To confirm whether ESAT-6 gene was present in the plasmid Pmrlb.7, PCR analysis was performed, which confirmed the presence of the gene indicated by the PCR product of 300 bp (data not shown). The BL21 *Escherichia coli* were transformed using Pmrlb.7 plasmids, and ESAT-6 protein was induced with the help of IPTG. ESAT-6 protein from *E. coli* cell lysates were purified by IMAC column as described in our earlier publication (20); ESAT-6-bearing exosomes (ESAT-6-Exo) were lysed and subjected to Western blot analysis (**Supplementary Figure 1A**). The band intensity analysis suggests approximately 69% encapsulation of ESAT-6 protein; this study helped control accurate vaccination doses in mice. Additionally, the Western blot analysis demonstrated the presence of exosome surface markers such as CD63, CD81, Tsg101, and CD9 on the isolated EVs (**Figure 1F**). Exosome markers, such as CD63, CD81, and TSG101, showed increased levels in the Rv-Exo sample, which typically indicates a higher release of exosomes from *M.tb.*-infected cells. We also evaluated the exosomes for calnexin, a negative marker, to determine the purity of exosomes. The absence of calnexin in both exosomes confirmed the purity of the samples (**Figure 1F**).

### Exosome efficiently delivers antigen via cytosolic and endocytic modes

Exosomes enhance antigen processing and presentation through distinct mechanisms based on their mode of entry into macrophages. To evaluate the antigen delivery mechanism, Rv-Exo were incubated with J774 macrophages for 10 min and 1 h. The results showed fusion of labeled blue fluorescent protein colored exosomes with the cell membrane, suggestive of cargo release into the cytoplasm (**Figure 1G**, enlarged panels, red arrows). Intriguingly, endocytosis can also be seen on the membrane and inside the cytoplasm at 10 min as well as 1 h post-incubation, suggestive of endosomal delivery of exosome content (**Figure 1G**, enlarged panels, blue arrows). The cytosolic delivery through a fusion mechanism enables antigens to be processed through ubiquitination and proteasomal degradation machinery (acting as endogenous antigen), leading to antigen presentation on MHC class I molecules, facilitating cross-presentation to CD8^+^ T cells and eliciting cytotoxic immune responses. In contrast, exosomes are internalized via the endocytic pathway, as shown in **Figure 1G** (blue arrow), which leads to antigen presentation on MHC class II molecules to activate CD4^+^ T cells. These complementary mechanisms underscore the critical role of exosomes in bridging innate and adaptive immune responses by enabling antigen presentation through both MHC class I and class II pathways.

Together, these data suggest that the isolated EVs obtained by the ultracentrifugation method were in the size range of exosomes and were pure as well. The alveolar macrophage cell line used in this study to isolate exosomes was characterized by FACS analysis. Approximately 96.41% of cells were found to express high levels of CD11c, of which 17.51% were positive for F4/80. On the other hand, 94.06% of the population showed CD206 low phenotype (S1D). CD206 low and CD11c high and F4/80 low also confirm the cells as alveolar macrophages (AMΦ) (**Supplementary Figures 1B–E**).

### Rv-Exo proteins demonstrate the presence of both B- and T-cell antigenic determinants

The exosome vaccine candidates characterized for size and purity were subjected to liquid chromatography–mass spectrometry (LC–MS). The LC–MS data showed 143 proteins of *M.tb.* origin and 1,032 host proteins within exosomes isolated from *M.tb.*-infected alveolar macrophages (Rv-Exo). The intricate host–pathogen interplay during *M.tb.* infection emphasizes the roles of specific exosomal proteins. Additionally, the presence of *M.tb.* proteins in exosomes implies their potential implication in modulating the immune response and bolstering host defense mechanisms against *M.tb*. Considering exosomes’ pivotal roles in intercellular signaling and immune regulation, these findings suggest their involvement in shaping the immune response to *M.tb.* and may serve as valuable biomarkers for TB diagnosis or as targets for therapeutic interventions aimed at disrupting *M.tb.* pathogenesis. In this work, the protein sequence was submitted to predictive analysis using IEDB in order to discover possible B-cell epitopes (**Supplementary Table 1**) capable of eliciting humoral and T-cell epitopes capable of interacting with different MHC class I (**Supplementary Table 2**) and class II alleles (**Supplementary Table 3**). A total of 49 epitopes were predicted to bind MHC class I alleles, and 48 epitopes were predicted to bind MHC class II alleles; 46 B-cell epitopes were also predicted (**Supplementary Tables 1**–**3**). These peptides were selected based on their ability to achieve a predicted binding score below 1, indicating a high probability of binding to the respective MHC alleles. The epitopes identified through this study exhibited a high affinity for their respective MHC alleles, indicating strong potential as vaccine targets or diagnostic markers for TB. In conclusion, the bioinformatics analysis of pathogenic proteins present in Rv-Exo demonstrated the presence of antigenic determinants capable of provoking Th cells and cytotoxic T lymphocytic responses along with effective humoral responses.

### Rv-Exo contain T-cell antigens that are absent in the BCG genome

The proteomics analysis of Rv-Exo lysate revealed the presence of 143 proteins of the *M.tb.* H37Rv strain, but were conspicuously absent in the genome of the BCG strain. Approximately 46 B cell-associated (**Supplementary Table 1**), 49 Th-1-associated (**Supplementary Table 2**) and 48 Th-2-associated (**Supplementary Table 3**) antigens with a high number of epitopes were detected in Rv exosomes. Approximately 12 immunogenic proteins shown in **Supplementary Table 4** are of H37Rv origin and absent in BCG. This protein discrepancy highlights a potential genetic divergence between the BCG vaccine strain and the pathogenic *M.tb.* strain H37Rv. Antigenic analysis of 12 proteins depicts that those 8 proteins had an enormous number of MHC-I agrotope compatible epitopes, suggesting inducing a CTL response by Rv-Exo vaccination. Therefore, Rv-Exo could be used as a booster to supplement a strong CTL response. Overall, the presence of this protein within exosomes, which are pivotal mediators of intercellular communication and immune regulation, suggests its involvement in host–pathogen interactions and immune modulation during TB infection.

### Exosomes containing ESAT-6 and Rv-Exo induce the production of Th-1 immune responses

**Figure 2:**
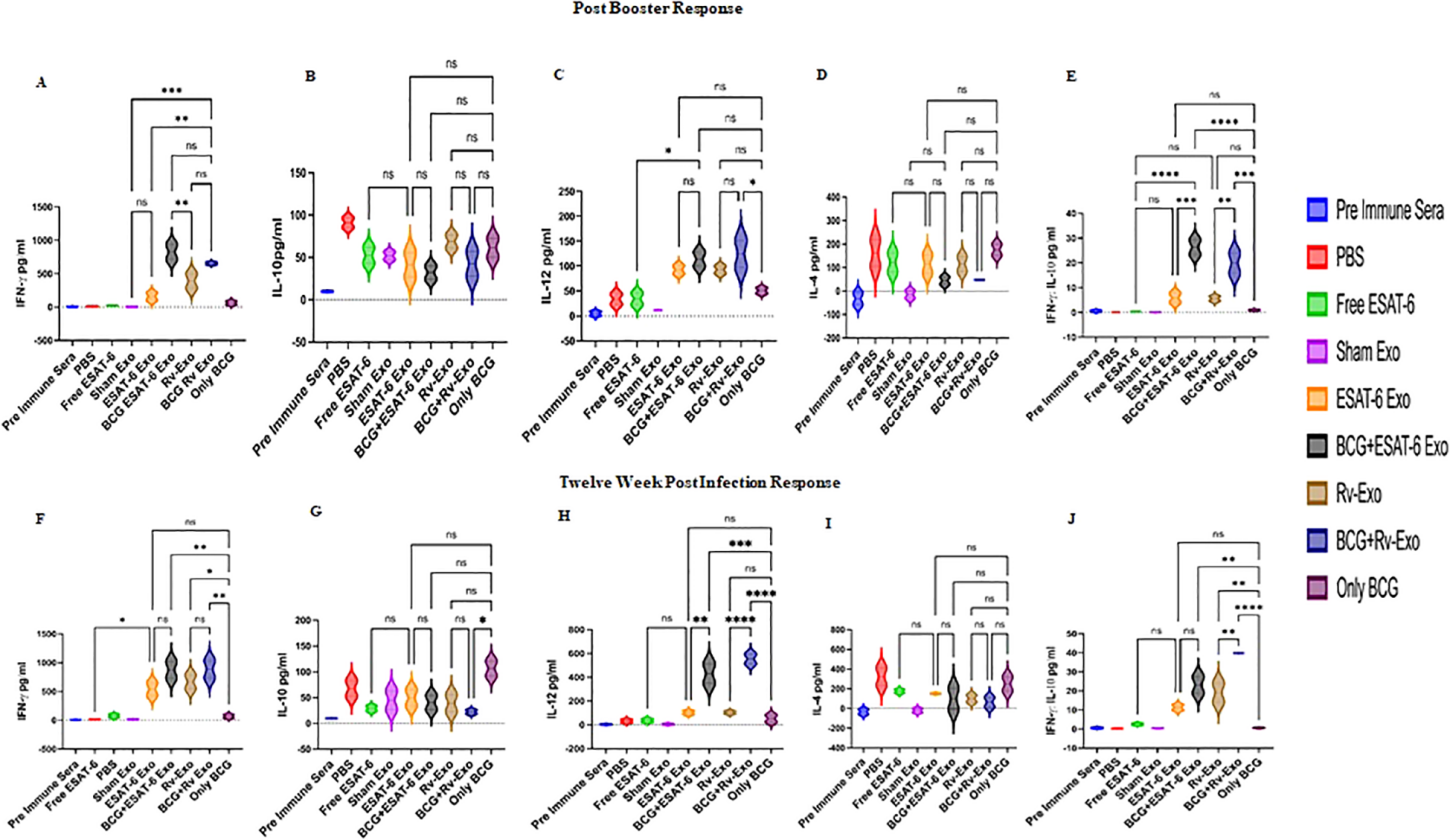
Cytokine immune response in serum, the *in vitro* system, and tissue samples. The Th-1 (IFN-γ and IL-12) and Th-2 (IL-4 and IL-10) cytokines were quantified in the serum of immunized animals using ELISA at 2 weeks post-booster: IFN-γ **(A)**, IL-10 **(B)**, IL-12 **(C)**, IL-4 **(D)**, and IFN-γ/IL-10 ratio **(E)**. Twelve weeks post-infection IFN-γ **(F)**, IL-10 **(G)**, IL-12 **(H)**, IL-4 **(I)**, and IFN-γ/IL-10 ratio **(J)**. All experiments were performed in triplicate, having three biological replicates in each experiment and data are presented here as the mean and standard error. Different vaccinated groups were compared to determine the statistical significance of the data using ANOVA with the Tukey test analysis with *p* < 0.05 (*), *p* < 0.01 (**), and *p* < 0.001 (***) level of significance.

Here, we have examined the immunogenic potential of exosomes containing the antigens ESAT-6 (ESAT-6 Exo) and H37Rv-infected alveolar macrophage-derived exosomes (Rv-Exo) as an antigen delivery system. Serum samples from various immunized cohorts were assayed for Th-1 (IFN-γ and IL-12) and Th-2 (IL-4 and IL-10) cytokines using sandwich ELISA to assess the Th-1/Th-2 ratio. Exosome-based vaccine candidates, i.e., ESAT-6 Exo and Rv-Exo, and their booster in BCG-immunized groups and BCG alone were examined. Exosome-encapsulated vaccine candidates for ESAT-6 Exo and Rv-Exo induce significantly elevated levels of Th-1 cytokines at various time points (**Figures 2A–J**). The Th-1 cytokine (IFN-γ and IL-12) levels are higher in the BCG group boosted by ESAT-6 Exo and Rv-Exo compared to the BCG-alone group at post-booster time points (**Figures 2A, C**). On the other hand, no significant difference was observed in the Th-2 (IL-4 and IL-10) response at any time points at post-booster. However, the IL-4 level was lower but not significantly in vaccinated groups compared to the sham exosome (PBS) group (**Figure 2D**). The Th-2 cytokine IL-10 was significantly high in the PBS group (*p* > 0.001) compared to ESAT-6 and Rv-Exo booster groups of BCG at the post-booster time point (**Figure 2B**). The expression level of Th-1/Th-2 cytokine ratio was 28 and 21 times higher in BCG+ESAT Exo and BCG Rv-Exo groups as compared to the BCG-alone group at the post-booster time point (**Figure 2E**); however, discernible elevation was observed across all groups at two weeks post-challenge (TWPC), indicative of *M.tb.* infection and likely representing the onset of the disease (**Figure 2**). At TWPC, the serum of vaccinated animals was evaluated for IFN-γ and IL-12 (Th-1 cytokines) as well as IL-4 and IL-10 (Th-2 cytokines). The comparative analysis showed elevated IFN-γ after booster in all groups. The vaccine group i-e ESAT-6 Exo (156 pg/mL PB vs. 570.15 pg/mL TWPC), Rv-Exo (390 pg/mL PB vs. 665.5 pg/mL TWPC), and their BCG booster counterparts showed 8- to 10-fold elevation compared to BCG alone (**Figure 2F**). Similarly, the IL-12 level in the ESAT6-Exo and Rv-Exo vaccinated groups were elevated five- to sixfold compared to the post-booster time point (**Figure 2H**). This sustained increase in IL-12 at 12 weeks post-challenge signifies protective immune response against *M.tb.* in immunized animals. When we compared Th-2 cytokine, IL4 production at post-booster and TWPC time points, the PBS group showed various fold elevated IL-4 production at TWPC compared to all vaccinated groups; it may be due to the onset of disease (**Figures 2D, I**). In contrast, the IL-10 levels were increased in PBS (90.5 pg/mL Pb vs. 67.7 pg/mL TWPC) and vaccinated groups, ESAT-6 Exo (41 pg/mL Pb vs. 49.5 pg/mL TWPC), Rv-Exo (68.5 pg/mL PB vs. 50 pg/mL TWPC), and BCG alone (61 pg/mL Pb vs. 120 pg/mL TWPC). Interestingly, the BCG also did not show much increase in IL-4 level (175.5 pg/mL Pb vs. 338 pg/mL TWPC). However, when the IL-10 level in post-challenge sample was compared between groups, the PBS group showed the highest IL-10 level (67 pg/mL) in comparison to other vaccinated groups, again proving the notion that *M.tb.* pathogenesis is having more impact on the PBS group. The BCG group also showed a heightened level of IL-10 (106.588 pg/mL) compared to its booster counterparts i-e BCG –ESAT-6 Exo (20 pg/mL) and BCG Rv-Exo (40 pg/mL). This finding remains in line with the earlier literature that suggests BCG strongly induces IL-10 response (**Figure 2G**).

After the evaluation of serum from immunized animals for Th-1 and Th-2 cytokines, we examined *M.tb.*-specific immune response. Equal numbers of splenocytes of control and vaccinated animals were either left untreated or activated using PPD 25, 50, or 100 µg/mL for 72 h, and culture supernatant was subjected to ELISA for IFN-γ (**Figure S3A**) and IL-10 (**Figure S3B**). As the antigen dose increased, the IFN-γ and IL-10 levels were also increased in the BCG+ESAT-6 Exo- and Rv-Exo-immunized groups (**Figures S3A, B**). The production of Th-1 and Th-2 cytokine exhibits *M.tb.*-specific immune responses in splenocytes of immunized animals. The cytokine profile distinctly illustrates *M.tb.* antigen-specific bearing exosome vaccine candidates; ESAT-6 Exo and Rv-Exo induce significantly elevated levels of Th-1 cytokines at post-infection (**Figure S3C**). It is also observed that ESAT-6 Exo- and Rv-Exo-boosted BCG groups demonstrated elevated dose-dependent cytokine levels. Overall, their results suggest that the *M.tb*. antigen-bearing exosomes not only induce Th-1-biased immune response but also efficiently boost *M.tb.*-specific Th-1 skewed response in BCG-immunized animals both in serum and in splenocyte culture supernatant upon activation by PPD (**Figure 2** and **Figure S3**).

### Immunohistochemistry analysis demonstrates Th-1 cytokine response in spleen and lung tissues

The microscopic examination of lung and spleen tissues stained for Th-1 (IFN-γ) and Th-2 (IL-10) cytokines revealed distinct patterns of immune response across the different groups. The control group showed minimal staining for both IFN-γ and IL-10, indicating baseline cytokine expression levels with no significant immune activation. In contrast, the BCG ESAT-6 Exo group exhibited intense staining for IFN-γ in both lung and spleen tissues, suggesting a strong Th-1-mediated immune response (**Figure S4A**), while IL-10 staining was moderate, indicating a balanced immune response with a slight Th-1 dominance (**Figure S4B**). Similarly, the BCG+Rv-Exo group demonstrated pronounced IFN-γ staining, particularly in the lung tissues, highlighting a robust localized Th-1 response, with IL-10 staining present but less intense, suggesting a predominant Th-1 response with some regulatory Th-2 activity. The only BCG-alone group showed moderate IFN-γ staining in both lung and spleen tissues, indicating an effective but less pronounced Th-1 response compared to the exosome-treated groups, with slightly elevated IL-10 staining reflecting a balanced immune response with a tendency towards Th-1 dominance.

Overall, the tissue microscopic images demonstrate that BCG+ESAT-6 Exo and BCG+Rv-Exo treatments significantly enhance Th-1 (IFN-γ)-mediated immune responses in the lungs and spleen compared to the control and only BCG groups. The presence of Th-2 (IL-10) cytokines suggests a regulatory mechanism to balance the immune response, with a notable Th-1 bias in the exosome-treated groups. These findings underscore the potential of BCG exosome treatments in modulating immune responses more effectively than BCG alone.

### The administration of exosome-entrapped ESAT-6 vaccine candidates predominantly elicits IgG2a subtype antibodies in mice subjected to immunization

**Figure 3:**
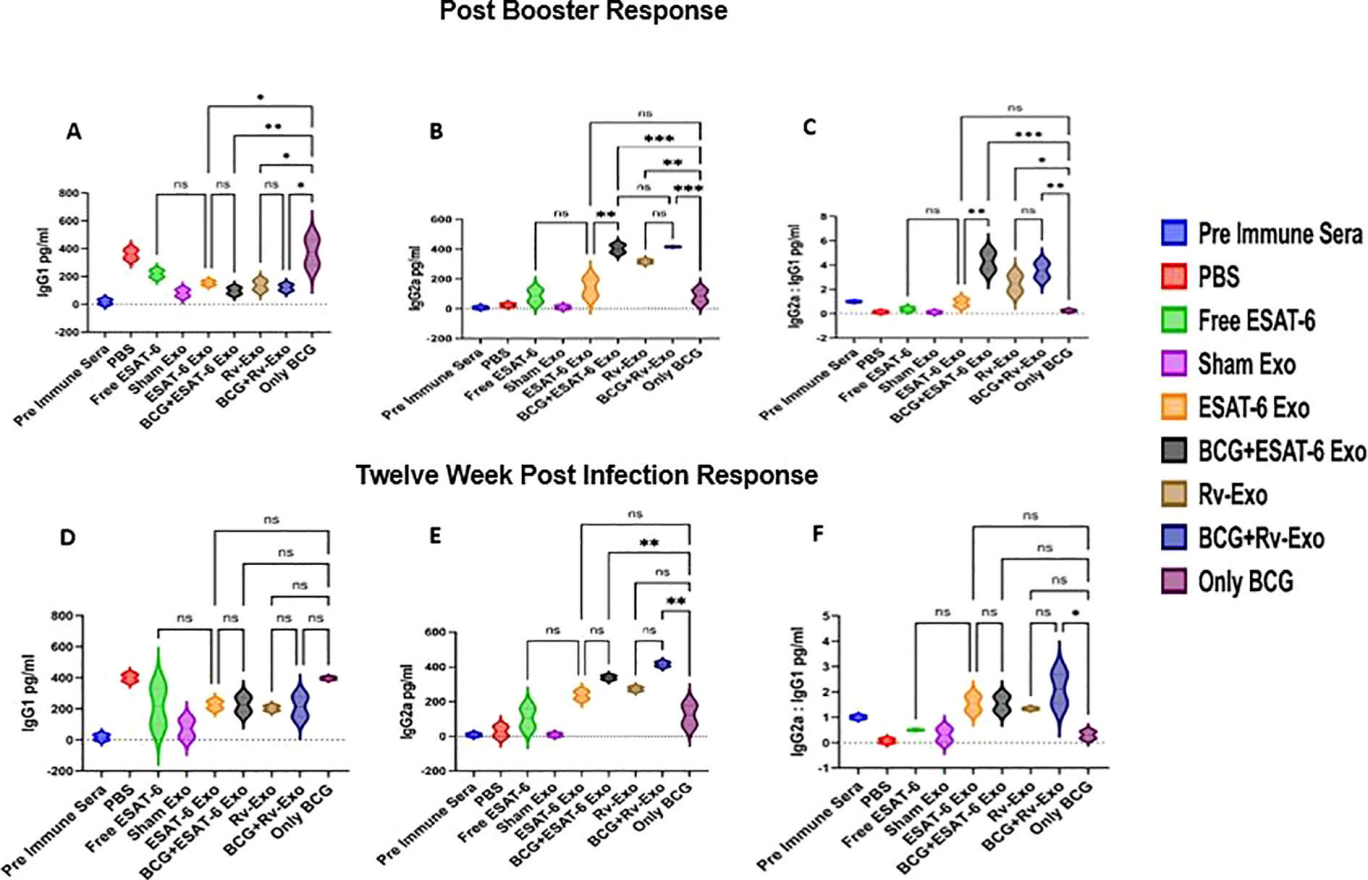
Humoral immune response in the serum of immunized animals: The animal serum was collected from all groups; subjected to ELISA for detecting IgG1 and IgG2a isotypes at post-booster IgG1 **(A)**, IgG2a **(B)**, and IgG2a/IgG1 ratio **(C)** and 12-week post infection IgG1 **(D)**, IgG2a **(E)**, and IgG2a/IgG1 ratio **(F)**; and analyzed. All experiments were performed in triplicate, having three biological replicates in each experiment and data are presented here as the mean and standard error. Different vaccinated groups were compared to determine the statistical significance of the data using ANOVA with the Tukey test analysis with *p* < 0.05 (*), *p* < 0.01 (**), and *p* < 0.001 (***) level of significance.

Apart from Th-1 and Th-2 cytokines, we examined the importance of IgG antibodies in Antibody-Dependent Cellular Cytotoxicity (ADCC), Antibody Dependent Phagocytosis (ADP), and CD4 activation. We evaluated IgG1 and IgG2a level isolated at post-booster and post-infection time points from various immunized groups using ELISA, and their ratio was also calculated. As illustrated in **Figure 3**, The expression of IgG1 levels is higher in our ESAT+Exo and BCG-alone group as compared to the vaccinated groups at post-booster time points (**Figure 3A**). Similarly, the expression IGg2a level is higher in our BCG+ESAT-6 Exo and BCG+Rv-Exo groups as compared to the only BCG groups (**Figure 3B**). There was a notable elevation in the IgG2a:IgG1 ratio at post-booster time points, with the BCG+ESAT-6 Exo and BCG+Rv-Exo groups exhibiting higher ratios of 10 and 7.5, respectively, compared to the BCG-alone group (**Figure 3C**). The post-booster results suggest that the BCG+ESAT-6 Exo and BCG+Rv-Exo groups elicit elevated Th-1-biased immune response and has the potential to educate the immune system for CTL response against *M.tb.* The post-booster IgG2a/IgG1 ratio measurements were 1.005 ± 0.05545 in pre-immune sera, 0.2555 ± 0.08300 for the BCG-alone group, 4.323 ± 0.6875 for the BCG+ESAT-6 Exo group, and 3.563 ± 0.5694 for the BCG+Rv-Exo group. At TWPC, the IgG1 level is higher in only BCG and BCG+Rv-Exo groups (**Figure 3D**) and the level of IgG2a is higher in the BCG+Rv-Exo group (**Figure 3E**). The IgG2a/IgG1 ratios were higher for the BCG+ESAT-6 Exo and BCG+Rv-Exo groups, 6 and 8.2, respectively, than the BCG-only group (**Figure 3F**). These findings suggest that the Rv-Exo and BCG+ESAT-6 Exo-immunized groups induce a more robust Th-1 response compared to other groups, including the BCG-alone cohort, with statistical significance (*p* < 0.001). The higher IgG2a:IgG1 ratio indicates a shift towards a Th-1-type immune response, crucial for combating intracellular pathogens like *M.tb.* Overall, we demonstrated that exosome-based vaccines, particularly those incorporating ESAT-6, significantly enhance the Th-1 immune response, making them promising candidates for improved TB vaccines.

### Increased pro- and anti-inflammatory cytokine expression in spleen and lymph nodes of immunized animals

**Figure 4:**
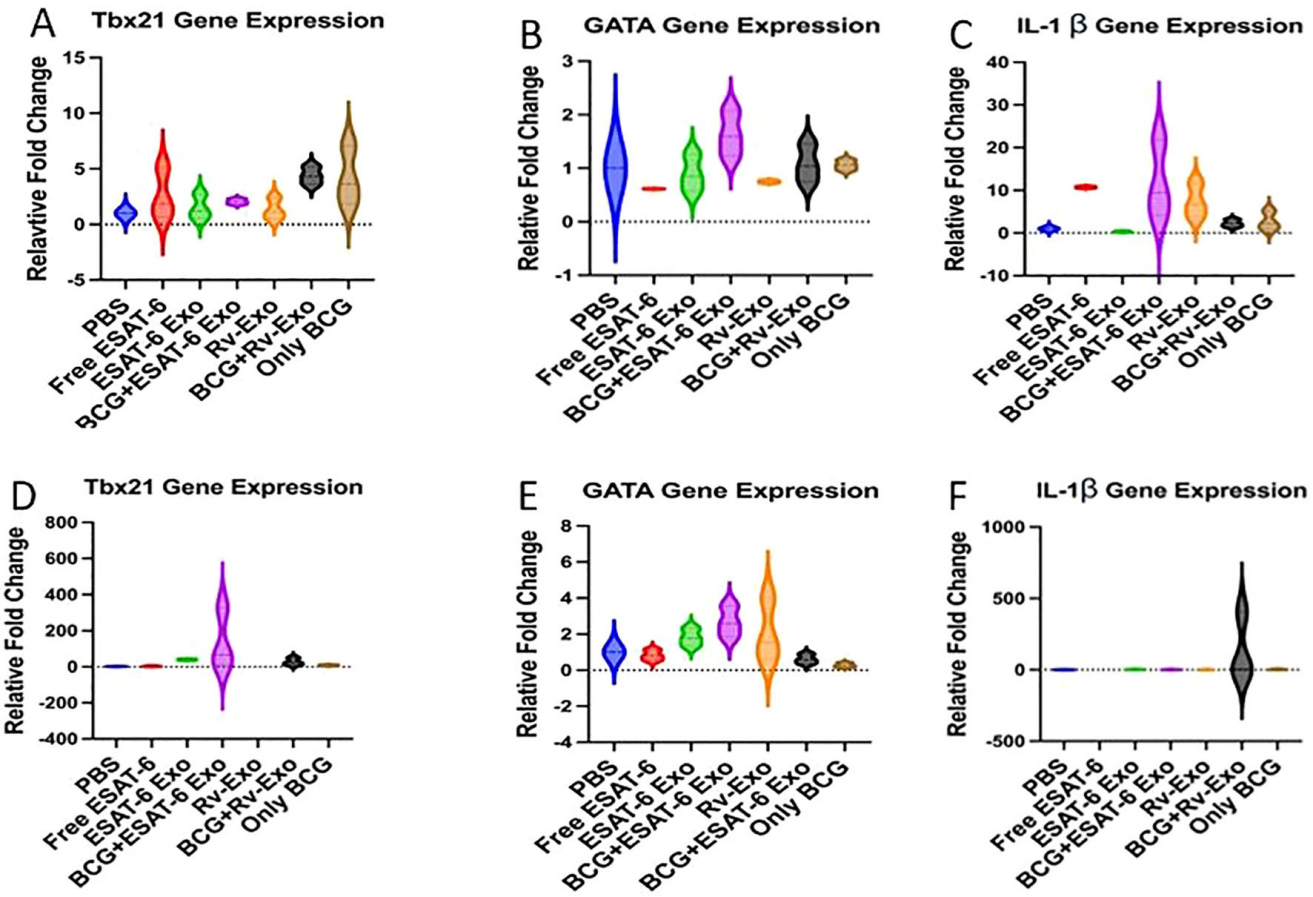
The Th-1 and Th-2 response-associated gene expression levels in the spleen and lymph node tissues of immunized animals. The lung and spleen tissues from various immunized group animals at twelve-week post infection were homogenize then total RNA was isolated using Trizol method. The RNA samples were analyzed for Tbx21 **(A)**, GATA3 **(B)**, and IL-1β **(C)** gene expression in spleen. Similarly, in the lymph node Tbx21 **(D)**, GATA3 **(E)**, and IL-1β **(F)** were analyzed. The significance of the data using ANOVA with the Tukey test analysis with *p*<.05 (*), *p*<.01(**), *p*<.001(***) level of significance. All experiments were conducted in three biological triplicates, and the data presented here represent the mean and standard error. Different vaccinated groups were compared to determine statistical.

The transcription factor, T-bet, is a hallmark of Th-1 immune response and mainly induces IFN-γ production. To examine *M.tb.*-specific type 1 immune response, we explored the signaling molecules. Quantitative PCR analysis was performed to assess the gene expression in the spleen and lymph nodes of various vaccinated groups at the TWPC. Our findings demonstrated heightened expression of Th-1 regulatory genes, Tbx21 in the spleen tissue of the Rv-Exo 1.5-fold and BCG+ESAT-Exo 1.6-fold group in comparison to the BCG-alone group (*p* < 0.001; **Figure 4A**). The level of Tbx21 gene expression was measured in control, BCG-alone, and BCG+Rv-Exo groups, with their respective values being 0.99 ± 0.002, 6.04 ± 0.349, and 9.22 ± 0.315 (**Figure 4A**). For the Th-2-regulatory gene GATA, expression was significantly upregulated in the spleen of BCG+Rv-Exo- and BCG+ESAT-6 Exo-immunized animals (**Figure 4B**). Interestingly, the pro-inflammatory cytokine IL-1 beta expression was found to be higher in only Rv-Exo and BCG+ESAT-6 Exo groups. In contrast, only BCG and BCG+Rv-Exo showed a similar response (**Figure 4C**). In contrast, in the lymph nodes, the expression of Th-1 immune response-related genes in vaccinated groups showed higher expression in comparison to the BCG-alone group (**Figure 4D**). Similarly, the expression of GATA-3 gene expression was higher in vaccinated groups as compared to the BCG-alone group (**Figure 4E**). IL-1 beta expression is higher in the ESAT-6 Exo and BCG+Rv-Exo group and BCG-alone group (**Figure 4F**). In conclusion, T-bet (Th-1 transcription factor) and GATA (Th-2 transcription factor) were elevated in immunized groups.

### CD3^+^/CD8^+^ and CD3^+^/CD8^+^ T and CD4^+^/CD45^+^ cell responses in vaccinated animals

**Figure 5:**
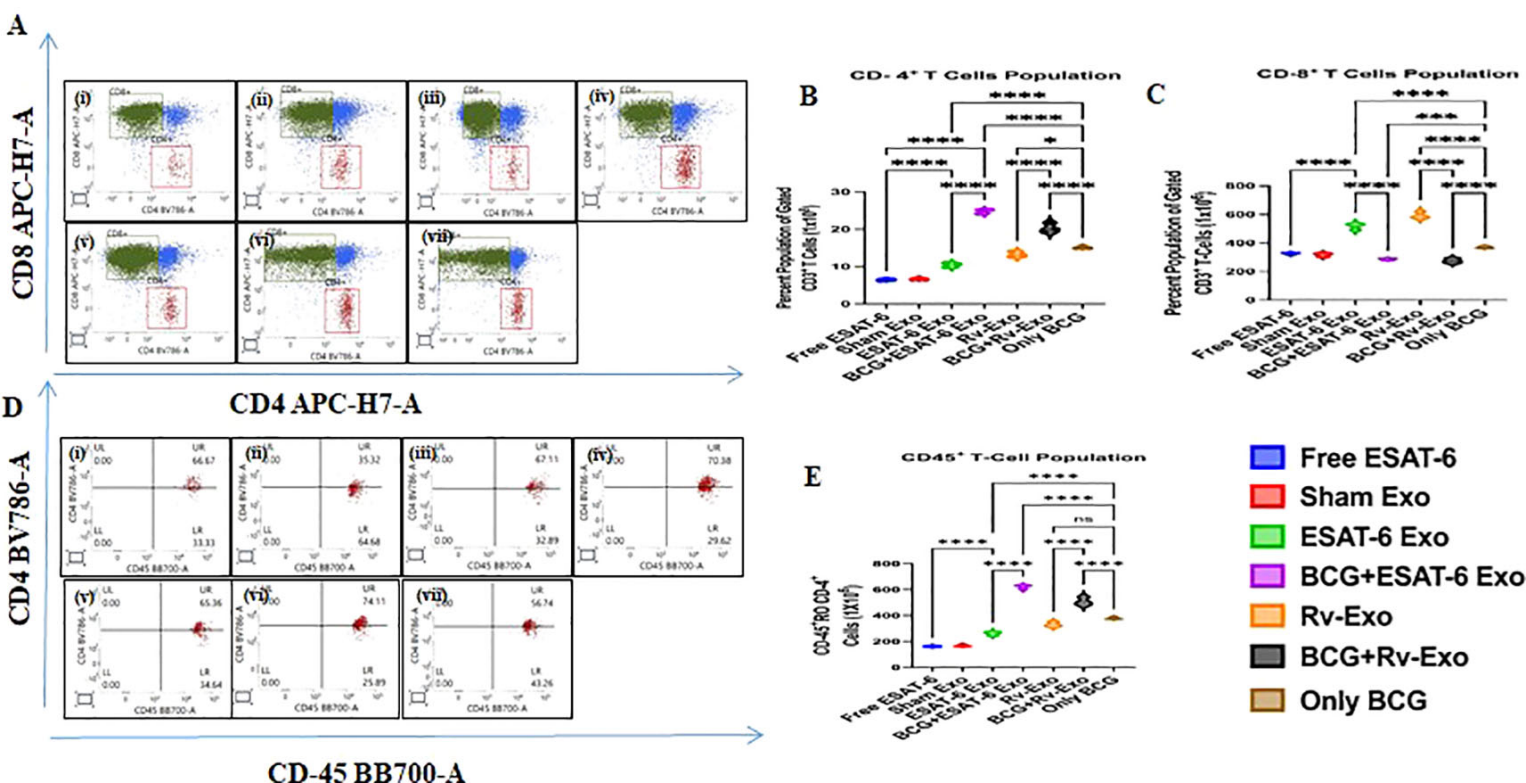
Evaluation of the T-cell population and activation in splenocytes of immunized animals. The splenocytes of Free ESAT-6 (i), Sham Exosome (ii), ESAT-6 Exo (iii), BCG+ESAT-6 Exo (iv), Rv-Exo (v), BCG+Rv–Exo (vi), and only BCG (vii) immunized animals isolated at 12 weeks post-challenge were subjected to FACS analysis. The CD3^+^ cells were gated and analyzed for CD4^+^ and CD8^+^ T lymphocyte population and presented as a dot plot **(A)**. The cells were enumerated and presented as a bar diagram for the CD4^+^**(B)** and **(C)** CD8^+^ T-cell population. Furthermore, the cell activation marker CD45 expression was analyzed on CD4^+^ T cells as can be seen in the dot plot **(D)**; cells were also enumerated and data are presented here as a bar diagram **(E)**. The data were analyzed with ANOVA test and are shown as the means (± SD) of two independent experiments with three biological replicates in each experiment. Different vaccinated groups were compared to determine the statistical significance of the data using ANOVA with the Tukey test analysis with *p* < 0.05 (*), *p* < 0.01 (**), and *p* < 0.001 (***) level of significance.

Lymphocytes from the spleens of various immunized animal groups at TWPC were stained with antibodies conjugated with specific cell surface molecules as well as intracellular markers. The cells were stained with anti-CD3 (pan T cell marker), anti-CD-4 and anti-CD8 to enumerate CD4+ helper T cells and CD8+ T lymphocytes (**Figure 5A**; dot plot). The FACS analysis showed that the BCG+ESAT-6 Exo and BCG+Rv-Exo groups exhibited a 1.6- and 1.34-fold higher population of CD4^+^ T cells, compared to the BCG-alone group: BCG vs. BCG+Rv-Exo (*p* < 0.001) and BCG vs. BCG+ESAT-6 Exo (**Figure 5B**). The elevated CD4^+^ T-cell population in the BCG ESAT-6 Exo and BCG+Rv-Exo booster groups demonstrates proliferation, which is crucial for orchestrating the immune response by activating other immune cells. On the other hand, the ESAT-6 Exo and Rv-Exo formulation was particularly found to be effective at inducing a CD8^+^ T-cell population, which is essential for directly killing infected cells (**Figure 5C**). The percentage of CD8^+^ T cells is 1.57- and 1.6-fold higher in the vaccinated groups as compared to the only BCG groups, as shown in **Figure 5C**. The increases in CD8^+^ T cells in the ESAT-6 Exo group were highly significant (*p* < 0.001). Moreover, the CD4^+^/CD45^+^ T helper cell population was 1.25- and 1.5-fold higher in the BCG+Rv-Exo and BCG+ESAT-6 Exo groups, respectively (**Figures 5D, E**). These results highlight that exosome-based vaccine candidates are modulating the immune response, enhancing T cell-mediated immunity, and suggest that these vaccine candidates have the potential to tailor immune responses more effectively than traditional vaccines, which could lead to improved protection against TB.

### Th-1 and Th-2 cytokine expression in CD4^+^ T cells

**Figure 6:**
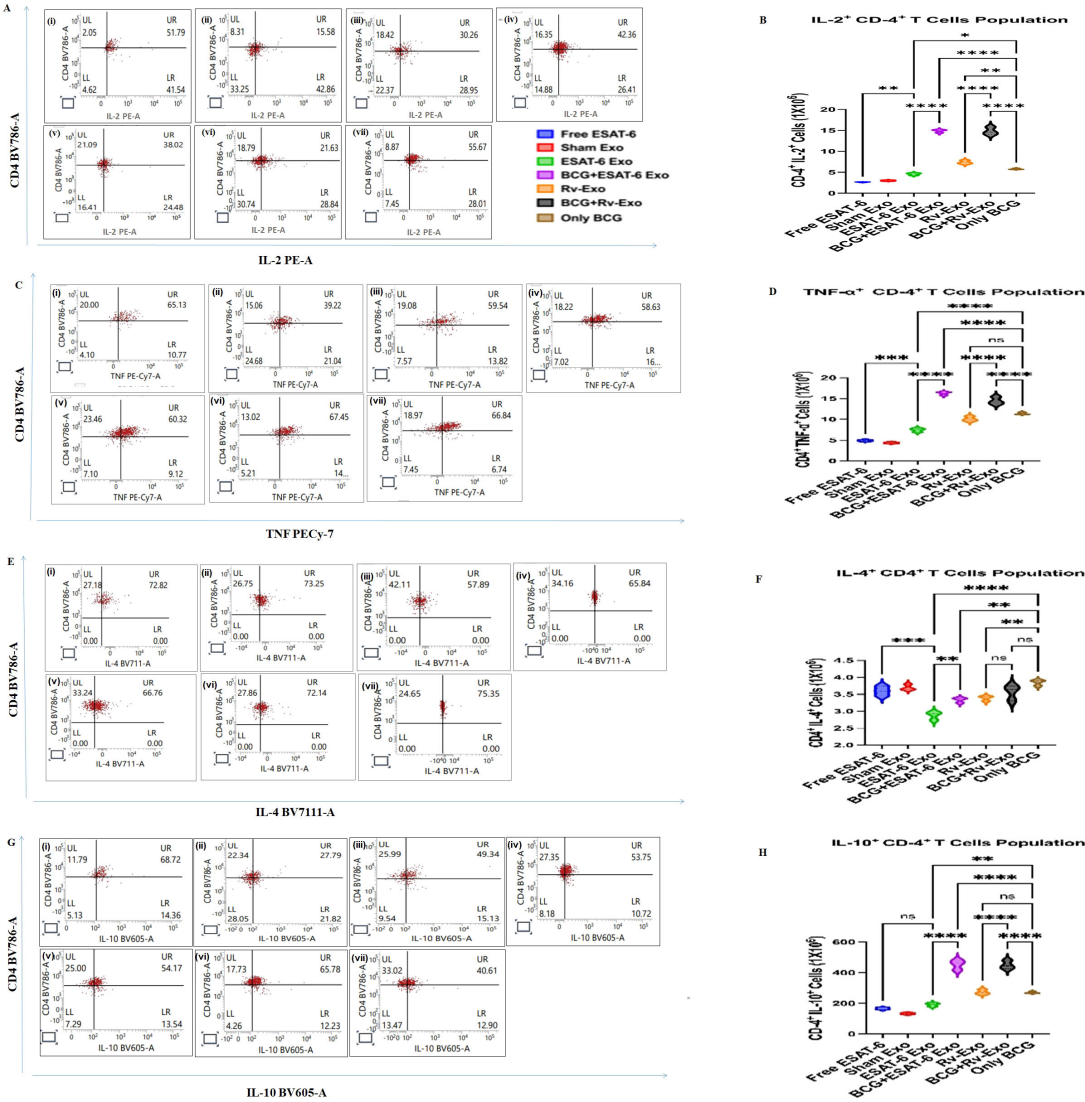
Evaluation of a functional T-cell population in splenocytes of immunized animals. The splenocytes of various vaccinated groups were isolated 12 weeks post-challenge and analyzed for Th-1 and Th-2 cytokine producing functional CD4^+^ T cells by FACS analysis. The representative dot plot of FACS depicts **(A)** CD4+IL-2, **(C)** CD4^+^TNFα^+^, **(E)** CD4^+^IL-4^+^, and **(G)** CD4^+^IL-10^+^ phenotypes of functional T cells. The total T-cell population of various groups was enumerated and presented here as a bar diagram; CD4+IL-2 **(B)**, CD4^+^TNFα^+^**(D)**, CD4^+^IL-4^+^**(F)**, and CD4^+^IL-10^+^ T cells **(H)**. The data were analyzed with ANOVA test and are shown as the means ( ± SD) of two independent experiments with three biological replicates in each experiment. Different vaccinated groups were compared to determine the statistical significance of the data using ANOVA with the Tukey test analysis with *p* < 0.05 (*), *p* < 0.01 (**), and *p* < 0.001 (***) levels.

The vaccinated groups elicited cytokines at various time points, suggestive of strong CD4^+^ T-cell response (**Figure 6**). Furthermore, we sought to investigate the CD4^+^ T cells’ involvement in cytokine production. To enumerate cytokine expression in a T-cell population, splenocytes of various immunized animal groups were stained for intracellular cytokines with conjugated antibodies specific for intracellular cytokines. The splenocytes were stained with α-CD3 α-CD4 antibodies along with intracellular Th-1 cytokines IL-2^+^ and TNF-α, and Th-2 cytokines IL-4 and IL-10 were stained to assess their intracellular expression levels. The results revealed that animals immunized with BCG and boosted with ESAT-6 and Rv-Exo exhibited significantly heightened expression of intracellular IL-12 at 12 weeks post-infection, as shown in **Figures 6A, B**. When compared, the percentage of CD4^+^ IL-2^+^ T cells are 2.6- and 2.5-fold higher in BCG+ESAT-6 Exo- and BCG+Rv-Exo-vaccinated groups than the BCG-alone group. Next, we examined the expression of another proinflammatory cytokine, TNF-α, in CD4^+^ T cells. TNF-α was found to be 1.4- and 1.2-fold higher in our BCG+ESAT-6 Exo- and BCG+Rv-Exo-vaccinated groups, respectively, when compared to the BCG-alone group at TWPC (**Figures 6C, D**). Additionally, we have also examined the expression of Th-2 cytokines IL-4 and IL-10 in CD4^+^ T cells. IL-4 was higher in animals immunized with the free ESAT-6, BCG-alone, and Sham Exo antigen compared to other vaccinated groups, ESAT-6 Exo and Rv-Exo (**Figures 6E, F**). IL-10-producing CD4^+^ T cells were found to be higher in animals immunized with BCG+ESAT-6 Exo and BCG+Rv-Exo compared to the other vaccinated groups (**Figures 6G, H**). The elevated levels of IL-2^+^ and TNF-α in the BCG+ESAT-6 Exo and BCG+Rv-Exo groups suggest a robust Th-1 response, which is critical for effective response against *M.tb.* The IL-10-producing cells show Th-2 response as well, which indicates a balanced immune response in the immunized animals. These findings highlight the enhanced capacity of exosome-based vaccine formulations to elicit strong Th-1 immune responses, which are essential for controlling infections.

### ESAT-6 Exo and Rv-Exo booster to BCG enhances central and effector memory response in CD4^+^ and CD8^+^ T cells

**Figure 7:**
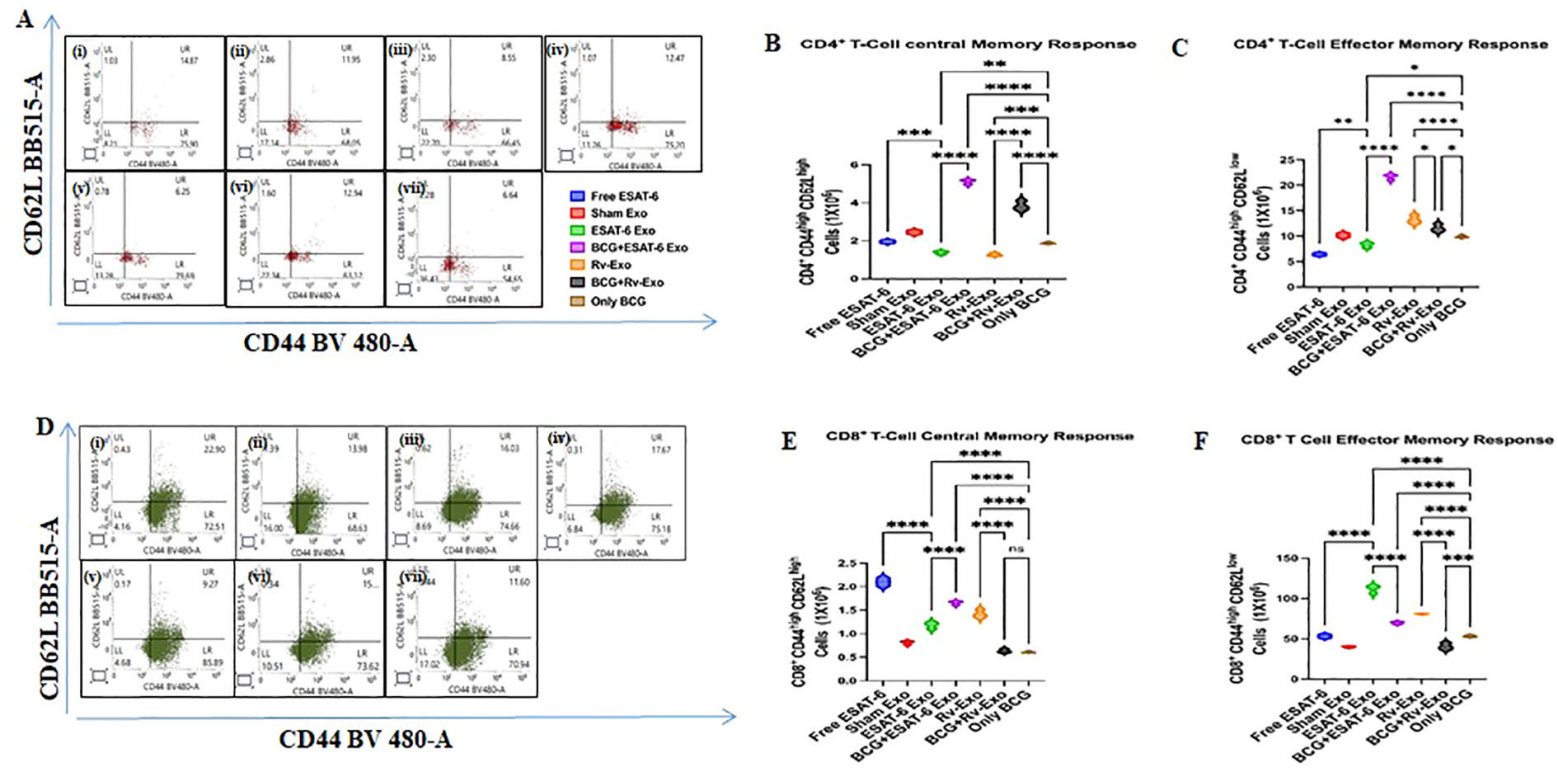
Evaluation of the effector and long-lasting central memory CD4^+^ T and CD8^+^ T-cell population in splenocytes of immunized animals. The splenocytes isolated from animals of various vaccinated groups at 12 weeks post-challenge were subjected to FACS analysis to enumerate the concurrent functional effector and long-term central memory markers. The representative dot plot images of CD4^+^ T cells **(A)** and their enumeration for CD4^+^CD44^high^CD62L^high^ (Central memory) **(B)** and CD4^+^CD44^high^CD62L^low^ (Effector memory) **(C)** are presented as a bar diagram. Similarly, the representative dot plot images of CD8^+^ T cells **(D)** and their enumeration for CD8^+^CD44^high^CD62L^high^ (Central memory) **(E)** and CD4^+^CD44^high^CD62L^low^ (Effector memory) **(F)** are presented as a bar diagram. Free ESAT-6, Sham Exosome, ESAT-6 Exo, BCG+ESAT-6 Exo, Rv-Exo, BCG+Rv-Exo, and only BCG populations were calculated. The data were analyzed with ANOVA test and are shown as the means (± SD) of two independent experiments with three biological replicates in each experiment. Different vaccinated groups were compared to determine the statistical significance of the data using ANOVA with the Tukey test analysis with *p* < 0.05 (*), *p* < 0.01 (**), and *p* < 0.001 (***) levels.

The success of any vaccine relies on the development of memory responses of lymphocytes. T-cell memory remains indispensable for intracellular pathogens like *M.tb.* In line with evaluating memory response, after evaluating CD4^+^ and CD8^+^ T-cell phenotypes, we asked if the in-house prepared vaccine candidates induce effector (CD44^high^CD62L^low^) or long-lasting central memory (CD44^high^CD62L^high^) population in the splenocytes of the immunized animals at TWPC time points (**Figure 7**). The FACS data suggested that when BCG-immunized animals were given a booster with ESAT-6 Exo or Rv-Exo, the central memory in CD4^+^ T cells was increased by 2.5- and 2-fold higher, respectively, in comparison to the BCG-alone group (**Figures 7A, B**). The evaluation of CD4^+^ T-cell effector memory (CD4^+^CD44^high^CD62L^low^) plays an instrumental role in fighting concurrent pathogens. The FACS data suggest that the ESAT-6 Exo and BCG-alone groups showed comparable cell populations with slightly increased response in BCG-alone groups. On the other hand, BCG ESAT-6 Exo showed a 2.2-fold elevated level of effector memory cell population (**Figures 7A, C**).

Lymphocytes from the spleens of immunized groups were stained with antibodies conjugated to specific cell surface markers, followed by FACS analysis. The cells were stained with CD3 antibodies to identify T cells, and subsequently with CD4 and CD8 markers to differentiate between CD4^+^ T cells and CD8^+^ T cells. Additionally, the cells were analyzed for CD44 and CD62L expression to assess central and effector memory T-cell responses in the vaccinated groups. Since the killing of infected cells to reduce the pathogen burden in vital organs relies on CTL response, we also examined CD8^+^ T-cell memory responses in the immunized groups. Interestingly, animals immunized with Rv-Exo showed a persistent central memory phenotype (CD44^high^CD62L^high^) (**Figure 7E**). In contrast, the CD8^+^ T cells from the ESAT-6 Exo-immunized group exhibited a strong effector memory phenotype (CD44^high^CD62L^low^) (**Figure 7F**). The effector memory phenotype (CD44^high^CD62L^low^) in CD8^+^ T cells was significantly higher in the BCG+ESAT-6 Exo groups compared to other groups, including the BCG-alone group (*p* < 0.001), as demonstrated in **Figure 7F**. In contrast, the BCG+Rv-Exo group showed reduced effector memory. Interestingly, Rv-Exo and ESAT-6 Exo also showed a higher population of CD8^+^ effector memory. These results suggest that different exosome formulations not only enhance targeted memory T-cell responses but also indicate a tailored immune memory profile. The BCG+ESAT-6 Exo formulation effectively boosts both CD4^+^ and CD8^+^ effector memory T cells, which are crucial for rapid immune responses upon re-exposure to pathogens. Meanwhile, the Rv-Exo formulation appears to support a central memory phenotype, which is important for long-term immunity. This differentiation in immune response profiles underscores the potential of exosome-based vaccines to provide more effective and lasting protection against infectious diseases compared to traditional vaccines.

### ESAT-6 and Rv-Exo elicit T-cell proliferation in a dose-dependent manner

**Figure 8:**
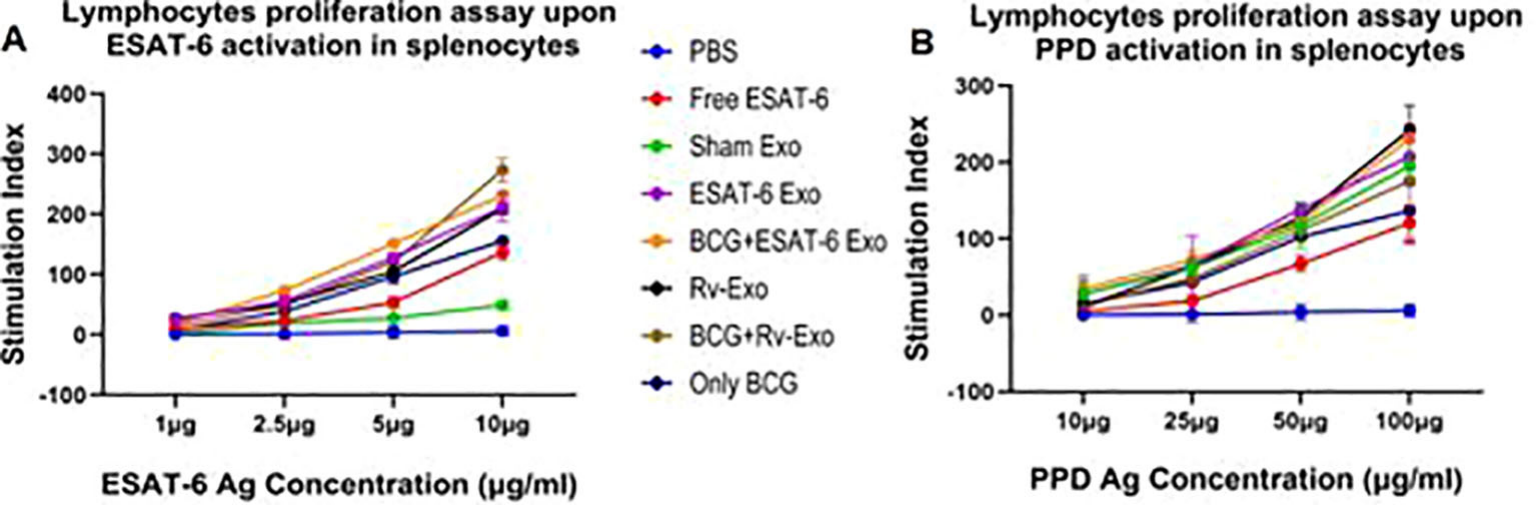
Antigen-dependent T-cell proliferation assay in splenocytes of various immunized groups. Splenocytes of immunized mice were isolated from PBS, Free ESAT-6, Sham Exosome, ESAT-6 Exo, BCG+ESAT-6 Exo, Rv-Exo, BCG+Rv-Exo, and only BCG groups at 12 weeks post infection and were incubated in 100 µL of media in flat-bottomed 96-well plates for 72 h at 37˚C in the presence of increasing amounts (1–10 µg/mL) of ESAT-6 Ag **(A)** and (10–100 µg/mL) of PPD Ag **(B)**; then, MTT assay was performed, and results were analyzed and presented here in the form of stimulation index. Different vaccinated groups were compared to determine the statistical significance of the data using ANOVA with the Tukey test analysis with *p* < 0.05 (*), *p* < 0.01 (**), and *p* < 0.001 (***) levels.

Splenocytes isolated from the immunized animals were seeded into 96-well plates. The splenocytes from both control and vaccinated animals were either left untreated or activated using ESAT-6 at concentrations of 1, 2.5, 5, and 10 µg/mL, as well as PPD at concentrations of 25, 50, or 100 µg/mL for 72 h at 37 °C (**Figures 8A, B**). The results showed that BCG+Rv-Exo significantly enhanced T-cell proliferation upon activation with the ESAT-6 antigen post-infection in a dose-dependent manner (**Figure 8A**). This enhanced proliferation is notably higher in the BCG+Rv-Exo and BCG+ESAT-6 Exo groups compared to groups treated with the free ESAT-6 antigen, BCG alone, and its physical mixture with sham-Exo. The observed increase in T-cell proliferation in the BCG+Rv-Exo and BCG+ESAT-6 Exo groups suggests a superior immunogenic response facilitated by the combination of BCG with Rv-Exo or ESAT-6 Exo. Furthermore, when the lymphocytes are activated with free PPD, a similar trend is observed where T-cell proliferation is significantly heightened in the BCG+Rv-Exo-immunized groups post-infection (**Figure 8B**). This indicates a robust and *M.tb.*-specific immune response triggered by the BCG+Rv-Exo immunization strategy. In sharp contrast, lymphocytes isolated from animals in the control group (immunized with PBS or sham exosomes) fail to induce substantial T-cell proliferation, even when exposed to higher antigen doses. This lack of significant proliferation in the control group underscores the efficacy of the BCG+Rv-Exo and BCG+ESAT-6 Exo immunization protocols in eliciting a potent T cell-mediated immune response. When the splenocytes were incubated, the control group showed the presence of macrophages and T cells, but they were not interacting as well as the macrophages, which are looking healthy (video 1). On the other hand, when the splenocytes of the BCG+Rv-Exo group were incubated, the T cells are surrounding the macrophages and killing them (video 2). These findings highlight the potential of exosome-mediated delivery in enhancing T-cell responses, offering promising avenues for improved TB vaccines.

### Oxygen burst response was elevated in immunized animals

**Figure 9:**
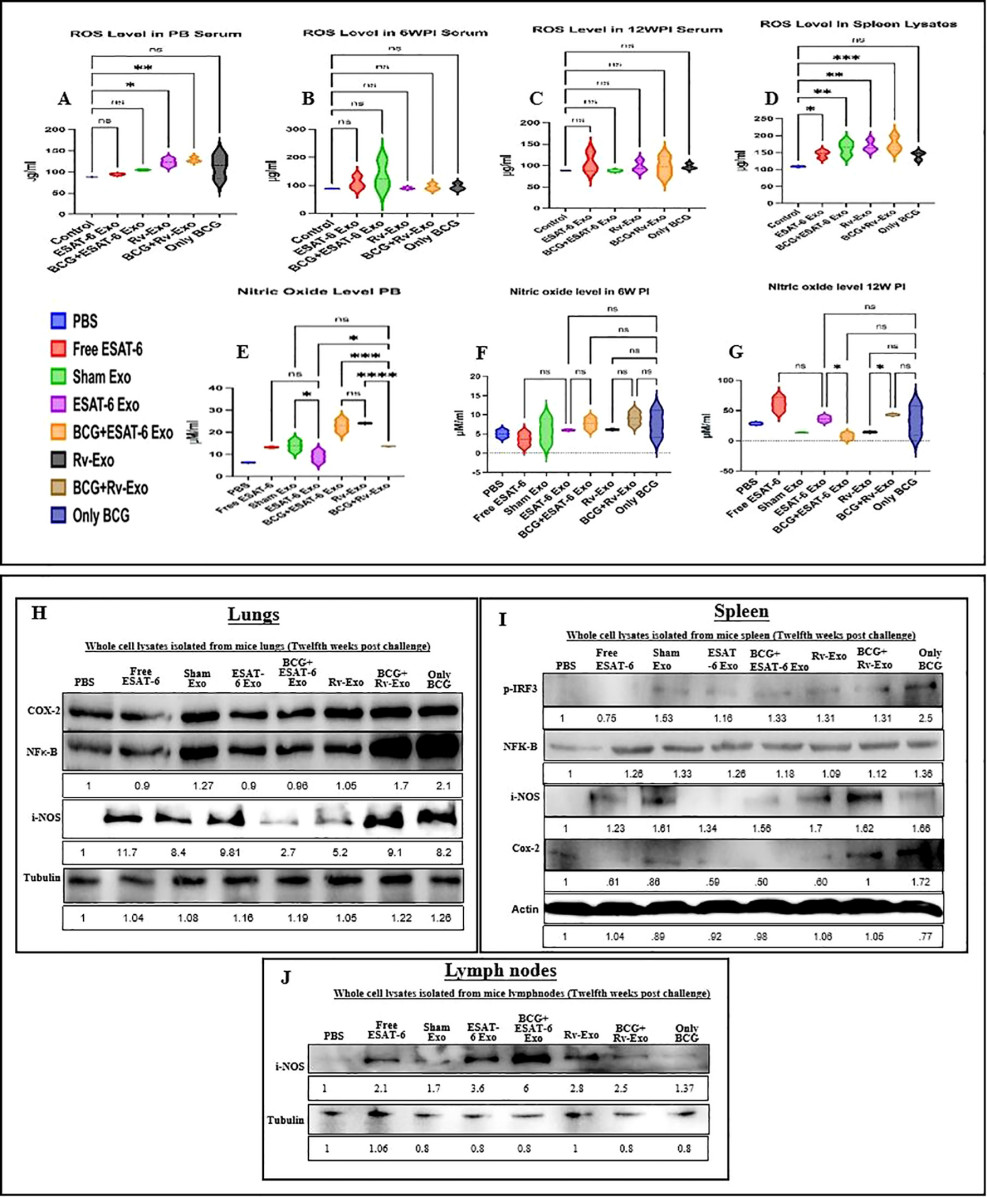
Detection of ROS and RNS in serum and iNOS in tissue samples of various groups of immunized mice. The serum from animals of various immunized groups were collected at post-booster, 6 weeks post infection, and 12 weeks post infection. The Griess reagent was used to detect nitric oxide level post-booster **(A)**, 6 weeks post infection **(B)**, and 12 weeks post infection **(C)**. The tissue from lungs **(H)**, spleen **(I)**, and lymph nodes **(J)** was isolated from various immunized groups on the 12th week post infection and protein lysates were prepared. Protein lysates were subjected to Western blot analysis to detect the expression levels of COX-2, iNOS, and NF-κB; β-actin was used as control. All experiments were done in triplicate, and data are presented here as the mean and standard error. Different vaccinated groups were compared to determine the statistical significance of the data using ANOVA with the Tukey test analysis with *p* < 0.05 (*), *p* < 0.01 (**), and *p* < 0.001 (***) levels.

The detection of NO and ROS levels in serum and whole cell lysates of spleen tissue from various immunized mice was conducted at three specific time points: post-booster, 6 weeks post-infection, and 12 weeks post-infection with *M.tb.* The results revealed that exosome-encapsulated vaccine candidates, BCG+ESAT-6 Exo and BCG+Rv-Exo, significantly elevated levels of NO and ROS in both serum and spleen tissue at all time points. Notably, ROS levels were higher in the BCG+ESAT-6 Exo (105 µg/mL) and BCG+Rv-Exo groups compared to the BCG-alone group post-booster (**Figure 9A**). Also, at 6 weeks post-infection, ROS levels were elevated in the ESAT-6 Exo group compared to the BCG-alone group (**Figure 9B**), and at 12 weeks post-infection, ROS levels remained high in the ESAT-6 Exo group compared to the BCG-alone group (**Figure 9C**). Upon comparative analysis of various groups, we found that ROS was elevated in comparison to post-booster in all groups like ESAT-6 Exo (130 µg/mL PB vs. 110 µg/mL TWPC) and Rv-Exo (130 µg/mL PB vs. 100 µg/mL TWPC). Additionally, ROS concentrations in splenic whole cell lysates were greater in the BCG+ESAT-6 Exo and BCG+Rv-Exo groups compared to the BCG-alone group at 12 weeks post-infection (**Figure 9D**). These results suggest that BCG ESAT-6 Exo and BCG Rv-Exo vaccines enhance ROS production, leading to a more robust and sustained immune response against TB. Similarly, NO levels were significantly elevated in the BCG+ESAT-6 Exo and BCG Rv-Exo groups compared to the BCG group post-booster (**Figure 9E**), and NO production remained higher in both the BCG ESAT-6 Exo and BCG Rv-Exo groups at 6 weeks (**Figure 9F**) and 12 weeks post-infection (**Figure 9G**). These results indicate that the BCG+ESAT-6 Exo and BCG+Rv-Exo vaccines enhance NO production, contributing to a more robust and sustained immune response against TB. These findings suggest that vaccination effectively enhances NO and ROS levels, contributing to the host defense mechanisms against TB infection. The exosome-based vaccine for TB modulates ROS and NO levels, enhancing the long-term immune response against *M.tb.* and protecting against tissue damage by inducing bactericidal activity, thus making it a promising candidate for TB prevention.

### Prophylactic efficacy of vaccine post-infection in the lungs, spleen, and lymph nodes of immunized mice

NF-κB regulates COX-2 and iNOS to facilitate inflammatory responses, to evaluate inflammatory signaling. The Western blot analysis was performed using whole cell lysates from lungs, spleen, and lymph nodes from various groups of immunized mice, and levels of COX-2, iNOS, and NF-κB were compared between groups. In **Figure 9H**, the whole cell lysate of lung tissue shows the expression of COX-2 protein, which is an inducible enzyme that plays a pivotal role in the inflammatory response during TB infection. Densitometry analysis was used to quantify the band intensities, which were normalized against tubulin and compared with the PBS control group. The results showed a significant increase in NF-κB expression across the vaccine candidates, with Rv-Exo showing a 1.05-fold increase and BCG+Rv-Exo showing a 1.8-fold increase. Interestingly, the BCG+Rv-Exo and BCG-alone groups showed a 1.7- and 2.1-fold increase, respectively. The expression of iNOS is increased, as shown by the fold change in the vaccinated groups: 9.81, 2.7, 5.2, and 9.1 for ESAT-6 Exo, BCG+ESAT-6 Exo, Rv-Exo, and BCG+Rv-Exo, respectively, as compared to the PBS group. The expression of Pirf-3, NF-κB, iNOS, Cox-2, and loading control actin was evaluated in the whole cell lysate of spleen tissue (**Figure 9I**). The results showed a significant increase in pIrf-3 expression across the vaccine candidates, with ESAT-6 Exo and BCG+ESAT-6 Exo showing a 1.16- and 1.33-fold increase, and Rv-Exo and BCG+Rv-Exo having a 1.33-fold increase. The expression of the iNOS molecule shows the increase in fold change of 1.34, 1.56, 1.7, and 1.62 in the vaccinated groups ESAT-6 Exo, BCG+ESAT-6 Exo, Rv-Exo, and BCG+Rv-Exo, respectively, when compared to the PBS group. The expression of the Cox-2 molecule shows an increase in fold change in the vaccinated group BCG+Rv-Exo (1.72) over the PBS group. The expression of iNOS in lymph nodes of different vaccinated animals was also increased as determined by the fold change of 5.1, 7.8, 3.9, and 3.4 in the intensity of bands in the vaccinated groups ESAT-6 Exo, BCG+ESAT-6-Exo, Rv-Exo, and BC+Rv-Exo, respectively, as compared to the PBS group (**Figure 9J**). These results are in line with the oxidative burst responses (**Figures 9A–G**). Overall, these findings suggest that both Rv-Exo and BCG+Rv-Exo may have a stronger immunogenic potential, making them promising candidates for further vaccine development.

### The exosome-based vaccine reduces bacterial burden in vital organs

**Figure 10:**
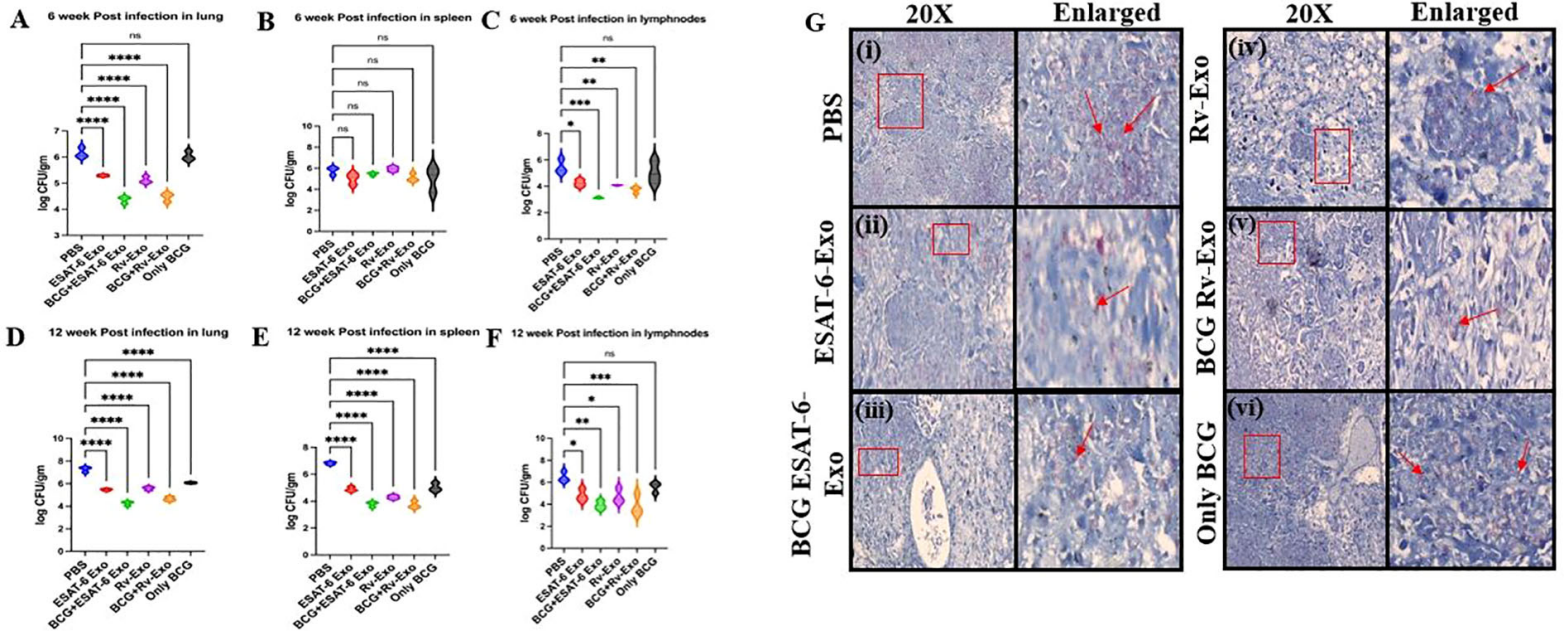
Evaluation of the prophylactic efficacy of various vaccines in immunized animals. Two weeks post-booster, various groups of immunized mice were aerosol challenged with *M.tb.* H37Rv and mice were analyzed for bacterial burden in tissue homogenates of lungs **(A)**, spleen **(B)**, and lymph nodes **(C)** at 6 weeks and 12 weeks post-challenge. The tissue homogenates were cultured to quantify colony-forming units (CFUs) at 6 and 12 weeks post-challenge. Results are expressed as mean values with standard deviation (± SD), based on three independent experiments with three biological replicates. Statistical analysis was performed using ANOVA with the Tukey test, with significance levels set at *p* < 0.01 (**) and *p* < 0.001 (***). Additionally, histopathological analysis of lung specimens from different immunization groups was conducted. Lungs collected at 12 weeks post-challenge were fixed in formalin, sectioned, and stained with hematoxylin and eosin (H&E) **(G)**, as well as Ziehl–Nielsen stain for acid-fast bacilli, according to established protocols. The experimental groups included PBS **(A)**, ESAT-6 Exo **(B)**, BCG ESAT-6 Exo **(C)**, Rv-Exo **(D)**, BCG Rv-Exo **(E)**, and BCG alone **(F)**.

In certain cases, though immunization leads to a strong immune response, the prophylactic efficacy of the candidate vaccine remains compromised. To test the potential of our exosome-based vaccines, we have challenged the vaccinated animals with *M.tb.*, and the protective efficacy was evaluated based on their ability to reduce bacterial loads in the lungs, spleen, and lymph nodes of immunized mice. As shown in **Figure 10**, groups immunized with ESAT-6-Exo or Rv-Exo with BCG demonstrated a significant reduction in bacterial burden compared to the PBS or BCG-alone group. At 6 weeks post-challenge, BCG followed by ESAT-6-Exo and Rv-Exo vaccination provided protection when compared to the PBS group (*p* < 0.001) in the lungs (**Figure 10A**). In the case of spleen, ESAT-6-Exo and BCG+Rv-Exo vaccination reduce bacterial burden when compared to the PBS group (*p* < 0.001) (**Figure 10B**). Additionally, lymph nodes showed a reduction in spleen mycobacterial load in BCG+ESAT-Exo and BCG+Rv-Exo log_l0_1.7 ± 0.35 and 1.4 ± 0.1386, respectively, when compared to the PBS and only BCG group (*p* < 0.001) (**Figure 10C**). By TWPC, animals immunized with BCG followed by ESAT-6 Exo and Rv-Exo exhibited a mycobacterial load that was log_l0_1.71 ± 0.1028 and log_l0_1.56 ± 0.098 lower than that of the PBS group in the lungs (**Figure 10D**). Similarly, the spleen animals immunized with BCG followed by ESAT-6 Exo and Rv-Exo exhibited a mycobacterial load that was log_l0_1.80 ± 0.1040 and log_l0_1.82 ± 0.154 lower than that of the PBS and only BCG group (**Figure 10E**). In the case of lymph nodes, booster groups showed a reduction of bacterial load that was log_l0_1.64 ± 0.226 and log_l0_1.67 ± 0.52 lower than that of the PBS and only BCG group (**Figure 10F**). Notably, BCG+ESAT-Exo and BCG+Rv-Exo showed an increase in CFU count in the lungs at TWPC compared to the bacterial burden at 6 weeks’ post-challenge in all organs. The residual bacterial load data clearly establish the superiority of the booster in eliminating TB compared to other control immunized groups.

### Histopathology analysis suggests reduced tissue damage as well as bacterial burden in lungs of immunized animals

We investigated histopathological alterations and the presence of acid-fast bacilli in the lungs of infected mice to monitor tissue damage immune cell infiltration and overall disease progression (**Figure 10G**). In mice immunized with BCG followed by ESAT-6 Exo and Rv-Exo, acid-fast bacilli were observed in lung tissue at TWPC (**Figure 10G**). This indicates that BCG followed by ESAT-6 Exo and Rv-Exo confers superior protection against TB by reducing bacterial burden compared to BCG alone and PBS. These findings underscore the potential of booster vaccine candidates as more effective strategies against TB, evidenced by improved histopathological outcomes and a lower prevalence of acid-fast bacilli in lung tissues. The observed reduction in TB-associated lung pathology and bacterial load signifies a stronger prophylactic effect, suggesting that this novel vaccine formulation may elicit a more robust immune response and enhance protective efficacy against TB infection.

## Discussion

There are currently 17 TB vaccine candidates in various phases of clinical trial (23). These vaccine candidates fall under three broad categories: (1) recombinant BCG or other mycobacteria species, (2) viral vectors expressing various mycobacterial proteins, and (3) recombinant mycobacterial proteins in conjugation with robust adjuvants (24). Recent data indicate that the MVA85A does not provide efficacious protection when used as a booster vaccine in infants previously immunized with BCG (25). Early reports with limited evidence suggest that exosomes may offer a novel approach to TB vaccine development. However, comprehensive studies to establish exosomes as viable candidates against TB remain largely unexplored. To establish the exosome-based vaccine, we employed *M.tb.*-specific antigen-bearing Rv-Exo and ESAT-6 Exo, which are absent in BCG, to investigate for their efficacy either as an independent vaccine or as a BCG booster (**Graphical Abstract**).

The BCG vaccine lacks virulence factors containing T-cell epitopes due to the deletion of RD regions facing several significant challenges that limit its effectiveness. Its efficacy varies widely, ranging from 0% to 80%, influenced by geographic differences and pre-existing immunity from exposure to non-tuberculous mycobacteria, particularly showing lower effectiveness against pulmonary TB in adults (26). Additionally, the immunity provided by BCG wanes over time, typically lasting only 10–20 years, which raises concerns about long-term protection, especially as susceptibility increases in young adults (27, 28). We proposed that supplementing the deleted gene products of BCG could control TB. To this end, we used *M.tb.-*infected cell-derived exosomes possessing *M.tb.* antigens that were absent in BCG as potential vaccine candidate either alone or as a BCG booster to amplify its immune prophylactic potency.

In previous studies, CFP-incubated RAW.2647 macrophage cell-derived exosomes were used as vaccine candidates (29). In another study, the exosomes isolated from *M.tb.*-infected macrophages have shown only immune modulatory activity (30). Here, we harvested exosomes from H37Rv-infected alveolar macrophages culture supernatant. Interestingly, those exosomes were found to carry 12 unique *M.tb.* proteins harboring T-cell antigens, namely, few Rv3887c and Rv1707, which were absent in BCG (**Supplementary Table 4**). Hence, Rv-Exo could supplement BCG-induced immunity to boost its efficacy. Previous studies suggested that APC-derived exosomes expressing antigen-bearing MHC-I or MHC-II molecules directly interacted with CD8 and CD4^+^ T cells. However, these exosomes were not capable of activating T cells directly unless they delivered the antigens to APCs, which in turn can activate T cells (31). The data of the present study suggest that exosomes derived from alveolar macrophages are capable of delivering the antigens in cytosol by fusion with macrophages. We speculate enhanced antigen presentation on MHC-1; hence, improved CTL response is expected. In contrast, exosomes were also found to be internalized by macrophages using an endocytosis mechanism, where antigens will be exogenous in nature (**Figure 1G**). The exogenous antigens should enhance antigen presentation on MHC-II to activate CD4^+^ T cells. Our data corroborate the previous studies where it has been shown that exosomes may deliver the content by fusion or by endocytosis (32).

In our study, a strong Th-1 polarization (IFN-γ:IL-10) was detected in the BCG+ESAT-6 Exo and BCG+Rv-Exo groups, indicating effective cell-mediated immunity compared to BCG alone at post-booster as well as post-challenge time points (**Figure 2**). IFN-γ-producing Th-1 cells typically drive the production of IgG2a antibodies. These findings underscore the importance of T cell-mediated immune responses in shaping antibody profiles and enhancing vaccine efficacy, especially in TB, where a robust Th-1 response is critical for protection. The *M.tb.* antigen-bearing exosomes have emerged as promising adjuvants in vaccine development due to their ability to promote effective antigen delivery and presentation to immune cells.

Our data suggest that an immunization protocol employing *M.tb.*-specific antigen-bearing exosomes, Rv-Exo, ESAT-6 Exo, and their BCG booster counterparts elevated IFN-γ level and increased IFN-γ:IL-10 ratio and IgG2a:IgG1 ratio at post-booster as well as post-challenge in immunized animals, suggestive of Th-1-biased immune response (**Figure 3**).

The histopathological analysis of lung tissues from mice immunized with BCG followed by ESAT-6 Exo and Rv-Exo reveals substantial improvements in disease outcomes relative to BCG alone or PBS control (**Figure 10G**). The marked reduction in acid-fast bacilli within vital organs, i.e., lung, spleen, and lymph nodes, indicates enhanced control of *M.tb.* infection. This reduced bacterial burden, along with diminished lung tissue damage, suggests improved prophylactic efficacy of Rv-Exo and ESAT-6 Exo booster groups compared to BCG alone (**Figure 10G**). These findings underscore the potential of exosome-based booster vaccines to overcome the limitations of BCG.

Furthermore, TBx21 upregulation indicates effective Th-1 polarization in lungs and spleen tissues of the BCG+ESAT-6 Exo and BCG+Rv-Exo-immunized mice (**Figure 4**). The Th-1 cytokine levels in serum of immunized animals correspond with Tbet and GATA gene expression profile.

The splenocytes isolated from BCG-immunized animals boosted with Rv exosomes or ESAT-6 Exo showed a dose-dependent pattern of T-cell proliferation (**Figure 8**). The higher antigen doses not only led to greater stimulation indices but also improved IFN-γ production, reflecting increased *M.tb.*-specific T-cell activation proportional to the antigen dosage, which was hampered in the BCG-alone group. The perturbed T-cell activation may be attributed to the lack of T-cell antigens in BCG, which was supplemented by injecting the T-cell antigen-bearing Rv-Exo and ESAT-6 Exo. The results suggest that Rv-Exo and ESAT-6 Exo are supplementing BCG with T-cell antigens. Furthermore, detailed investigation is required to decipher the key role of individual proteins present in the exosomes to identify the best antigens contributing to T-cell immunity in BCG-immunized mice boosted with Rv-Exo. It is well established that the T-cell immune response is indispensable to control intracellular pathogen including *M.tb*. In our study, elevated CD3^+^CD4^+^, CD3^+^CD8^+^, IL-2^+^CD4^+^, and TNF-α^+^CD4^+^ T cells were detected in splenocytes of Rv-Exo- and ESAT-6 Exo-boosted BCG groups (**Figures 5D, E**, **6A, D**). These findings indicate an enhanced Th-1-biased response. IL-2 plays a crucial role in T-cell proliferation and macrophage activation to control *M.tb.* infection In addition, the CD4^+^ T cells also demonstrated an elevated CD45RO expression, indicating the activation of effector memory T cells (**Figure 5E**).

BCG showed an increased IL-10-producing CD4^+^ T cell population compared to the free Ag vaccine candidate; however, when BCG-immunized animals were boosted with ESAT-6 Exo and Rv-Exo, the CD4^+^IL-10^+^ T-cell population was further increased. Eventually, enhanced IL-10 secretion by antigen-stimulated T cells was observed in ESAT-6 and Rv-Exo booster groups, compared to BCG alone (**Figure 6H**). This activation can lead to a more tolerogenic environment, which is particularly important in TB, where IL-10-mediated suppression may help to balance the immune response and prevent tissue damage (33).

The establishment of effector and central memories is essential to combat ongoing or future infections, respectively. In our study, CD4^+^ T cells derived from animals vaccinated with BCG+ESAT-6 Exo exhibited strong effector memory responses to *M.tb.* infection, characterized by high levels of CD44^+^ and low levels of CD62L expression on their surface (**Figures 7A, C**). Meanwhile, CD4^+^ T cells from animals vaccinated with both BCG+ESAT-6 Exo and BCG+Rv-Exo demonstrated robust long-term central memory response marked by high levels of CD44 and CD62L expressions on their surface (**Figures 7A, B**). In the case of CD8^+^ T cells, the effector memory was heightened in ESAT-6 Exo, BCG+ESAT-6 Exo, and Rv Exosome alone. Surprisingly, the splenocytes showed lower CD44^high^CD62L^high^CD8^+^ T-cell population in the Rv-Exo booster to BCG when analyzed for central memory, probably due to migration of these cells to lymph nodes (**Figures 7D, F**). Our vaccine candidates are focused on inducing central memory T-cell responses, which indicates that long-lasting and protective immunity can effectively combat future TB infections.

IFN-γ produced by T cells in response to *M.tb.* infection stimulates iNOS in macrophages, leading to increased production of NO (**Figure 9**), which resulted in the improved prophylactic efficacy against *M.tb*. challenge in vaccinated animals. These findings suggest that vaccination with BCG and booster with TB origin T-cell antigen-bearing vaccine candidates can prime the immune system to mount a stronger Th-1 response upon *M.tb.* infection as characterized by increased IFN-γ production.

In the context of *M.tb.* infection, there exists a dynamic interplay among inflammation signaling molecules, namely, NF-κB, iNOS, and COX-2, critical in combating the disease. NF-κB activation serves as a pivotal trigger in response to *M.tb.* infection, orchestrating the expression of pro-inflammatory genes (34–36). This activation often leads to the upregulation of iNOS, prompting the production of NO as a part of the host’s antimicrobial defense. However, excessive NO production can further stimulate NF-κB and induce COX-2 expression. COX-2, in turn, contributes to inflammation through the synthesis of prostaglandins, which modulates the immune response against *M.tb*. Thus, NF-κB, iNOS, and COX-2 collectively regulate the inflammatory milieu during *M.tb.* infection, shaping the host immune response and influencing the outcome of the infection (**Figure 9**). Our findings show that *M.tb.* antigen-bearing exosome-based vaccines, BCG+ESAT-6 Exo and BCG+Rv-Exo, significantly enhance NO and ROS production in serum and spleen tissues of immunized mice, which was observed post-booster and at 6 weeks and 12 weeks post-challenge with *M.tb*. (**Figures 9A–G**). The elevated levels of NO and ROS in serum, lung, and spleen tissue lysates of vaccinated and *M.tb.* challenged animals demonstrate the vaccine’s effectiveness in enhancing the innate immune response to curb the bacterial population within the infected cells.

We further assessed the role of these exosome-encapsulated vaccines in limiting the mycobacterial burden in three vital organs, including lungs, spleen, and lymph nodes. The results indicate that only the BCG ESAT-6 Exo and BCG+Rv-Exo treatments led to a significant decrease in bacterial load in the lungs, spleen, and lymph nodes of immunized animals by week 6 post-challenge, with a further reduction observed by week 12. Conversely, the Free ESAT-6 and BCG-only groups exhibited a significantly higher bacterial burden both at weeks 6 and 12 (**Figure 10**). The Th-1 skewed serum cytokine levels were also evident by immunohistochemistry and cytokine production by splenocytes (**Figure 2**, **Supplementary Figures 3**, **4**), greater IgG2a:IgG1 ratio (**Figure 3**), heightened CD4 and CD8^+^ T cells’ effector and central memory (**Figure 7**), and increased RNS and ROS response, which are likely responsible for reduced bacterial burden (**Figure 9**). Together, these data suggest that *M.tb.* antigen-bearing exosome vaccines confer long-term protection against TB in mice (**Figures 10A–F**). The effectiveness of BCG-induced protective immunity against TB could be enhanced by the booster of Rv-Exo and ESAT-6 Exo candidate vaccines.

In summary, *M.tb.* antigen-bearing exosome-based vaccine candidates were capable of improving the immunity and prophylactic efficacy of BCG against TB in a murine model as evident by the above-discussed results. Exosomes offer significant advantages, such as biocompatibility, enhanced antigen presentation, and immunocompatibility with the immune system. Because of the adjuvant potential of exosomes, stronger innate immune responses are speculated, which can further lead to enhanced CD4^+^ T helper cells’ and CD8^+^ T cells’ (CTL) immune responses, providing long-lasting protection against pathogens. The exosome was found to partially restore the function of exhausted T cells (37). However, challenges remain in using exosomes as a vaccine candidate due to batch-to-batch variations, raising concerns about the consistency and reliability of exosome-based vaccines. This problem could be mitigated by antigen/multiple antigen encapsulation as we employed for ESAT-6 Exo. *M. bovis* infects humans through zoonotic transmission. Notably, the live attenuated form of *M. bovis*, i.e., BCG, remains avirulent due to the loss of various virulent factors including T-cell antigens. These virulent factors are present in wild-type *M. bovis* and *M.tb.*, which leads to reduced T-cell immunity in BCG-immunized individuals. The experiments, conducted in animals, in this study as well as earlier studies suggest that boosting BCG with T-cell antigens could improve the prophylactic efficacy of the BCG vaccine. Furthermore, we are conducting clinical experiments where PBMCs from patients with TB are sensitized using exosomes isolated from *M.tb.* H37Rv-infected THP-1, A549, or HEK293 cells or exosomes isolated from patients with TB, so that we could establish whether *M.tb.*-specific T-cell antigen-bearing exosomes could be utilized to boost BCG immune response and prophylactic efficacy in vaccinated individuals.

## Data Availability

The original contributions presented in the study are included in the article/**Supplementary Material**. Further inquiries can be directed to the corresponding author.

## Supplementary material

Supplementary Figure S1 The entrapped ESAT-6 exosome were subjected to characterize either uninfected by the western blot for ESAT-6 antibody, the exosome was isolated from uninfected Alveolar macrophages & ESAT-6 protein entrapped by Sonication method (S1A). Characterization of alveolar macrophages by analysis. Murine macrophages cells were distributed by forward scatter (FSC) and side scatter (SSC). Cells regarded as alveolar macrophages were enclosed hexagonal form (B&C). The gated fractions of MS-H a cell line of murine Alveolar Macrophages from mice were stained by (D) F4/80PE-A- and APC-Cy7-A-CD11c and (E) CD206 Alexa 647-A.

Supplementary Figure S2 Lungs and spleen were isolated from different immunized groups. Morphology **(A)** and weight analysis of lungs **(C)** and spleens **(D)** from immunized animals. Quantitative analysis of lung and spleen weight (mg) across various immunized groups. Statistical significance is indicated as ***(p < 0.001), **(p < 0.01), and ns. All experiments were performed in triplicates, and data are presented as mean ± standard error.

Supplementary Figure S3 The splenocytes of immunized animals of various groups at twelve weeks post infection were stimulated with PPD for 72 hours and splenocytes culture supernatants were collected to assess the IFN-γ **(A)** and IL-10 **(B)** cytokines and IFN-γ/IL-10 ratio was also calculated **(C)**.

Supplementary Figure S4 The immunohistochemically analysis of lung **(A)** and spleen **(B)** tissues from four distinct groups: **(A)** Control, **(B)** BCG+ESAT-6 Exo, **(C)** BCG+Rv-Exo, and **(D)** Only BCG were assessed using antibodies specific to Th-1 (IFN-γ) and Th-2 (IL-10) cytokines.

Supplementary Video S1 Videos show interaction of T cells with infected macrophages: Spleen were aseptically isolated form mice either left unimmunized (control) or immunized with BCG and boosted with Rv-Exo In video 1 (control) both macrophages and T cells are present but interaction between them was not detected. On the other hand, in video 2, it can be seen that infected macrophages are surrounded by activated T-cells and later in the video we found altered cell morphology due to interaction with CD8+ CTL cells, and some cells are also showing apoptotic bodies. The video was recorded using a 20X lens and captures 5 minutes of footage using EVOS M7000. The video has been compressed into 30 seconds.
