# Supplementary figures and images for: Mycobacterium tuberculosis antigen-containing exosomes reinforce BCG vaccine efficacy by augmenting long-term protection and memory response against experimental tuberculosis in BALB-C mice
Source: Front Immunol. 2026 Mar 13;17:1742207. doi: 10.3389/fimmu.2026.1742207 (PMC13021648; doi:10.3389/fimmu.2026.1742207)

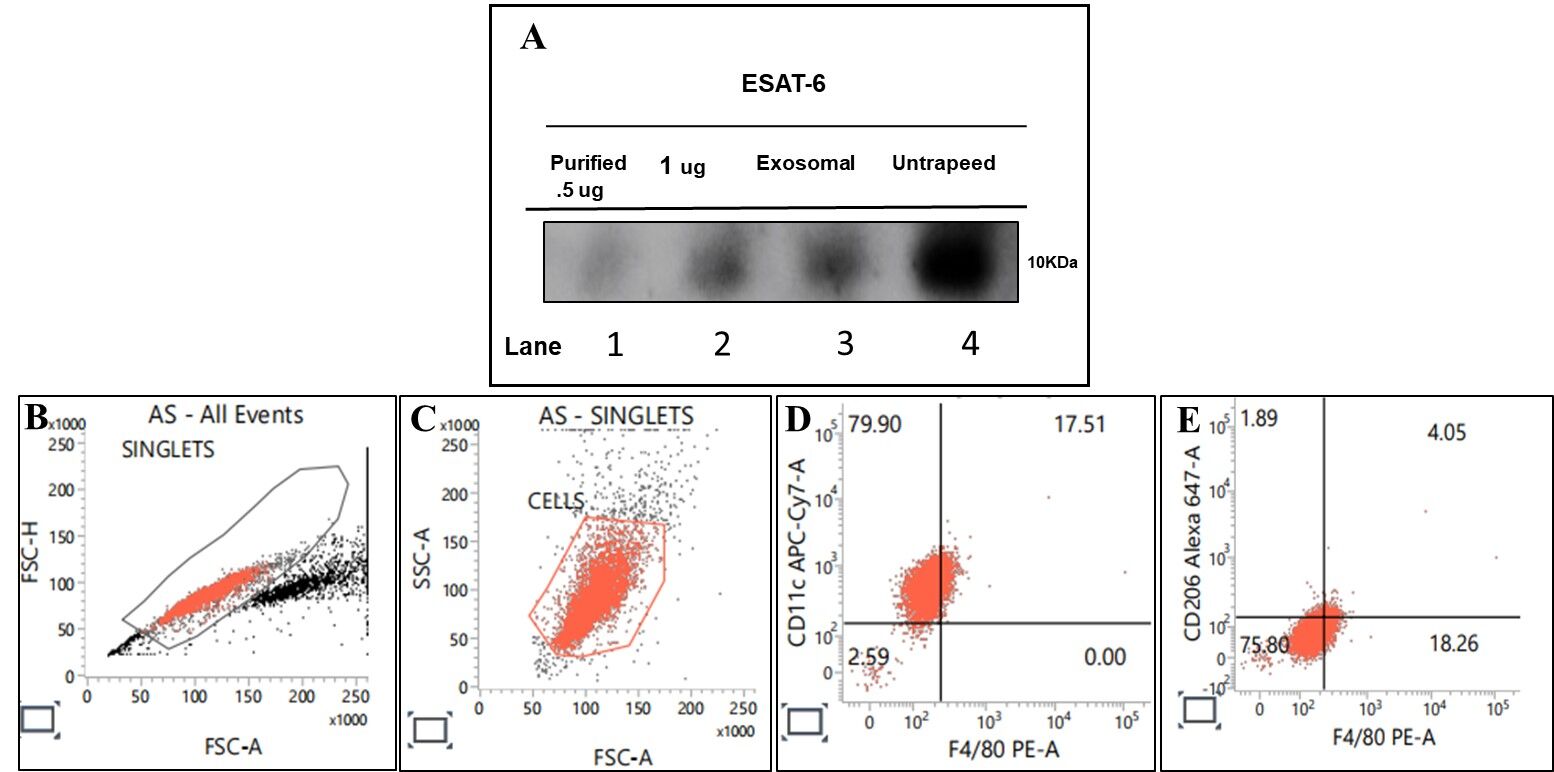

Supplement: Supplementary Figure S1 — The entrapped ESAT-6 exosome were subjected to characterize either uninfected by the western blot for ESAT-6 antibody, the exosome was isolated from uninfected Alveolar macrophages & ESAT-6 protein entrapped by Sonication method (S1A). Characterization of alveolar macrophages by analysis. Murine macrophages cells were distributed by forward scatter (FSC) and side scatter (SSC). Cells regarded as alveolar macrophages were enclosed hexagonal form (B&C). The gated fractions of MS-H a cell line of murine Alveolar Macrophages from mice were stained by (D) F4/80PE-A- and APC-Cy7-A-CD11c and (E) CD206 Alexa 647-A. [file Image1.jpeg]

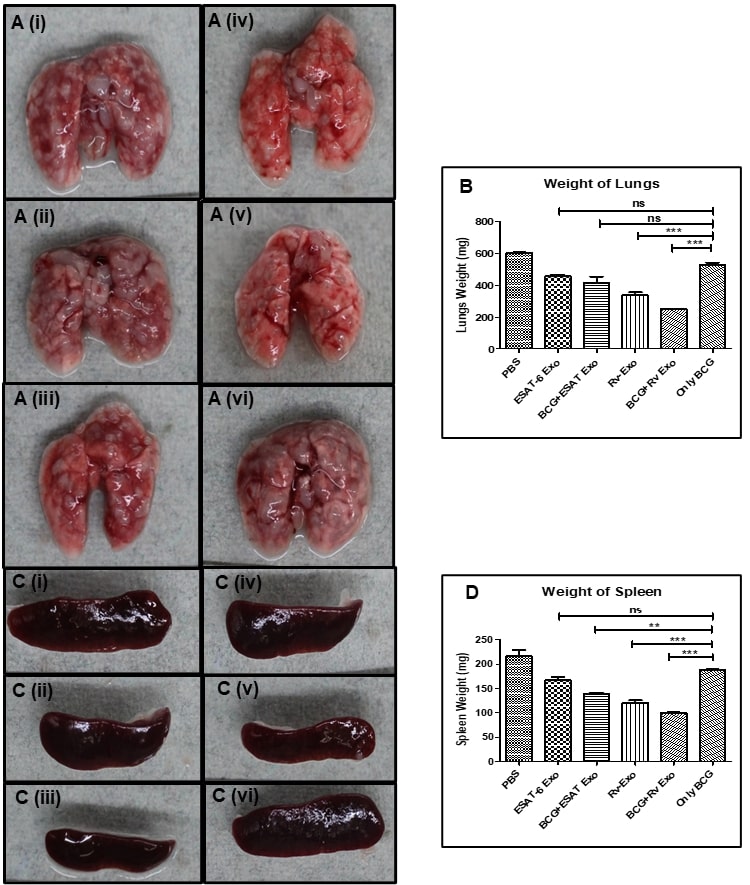

Supplement: Supplementary Figure S2 — Lungs and spleen were isolated from different immunized groups. Morphology (A) and weight analysis of lungs (C) and spleens (D) from immunized animals. Quantitative analysis of lung and spleen weight (mg) across various immunized groups. Statistical significance is indicated as ***(p < 0.001), **(p < 0.01), and ns. All experiments were performed in triplicates, and data are presented as mean ± standard error. [file Image2.jpeg]

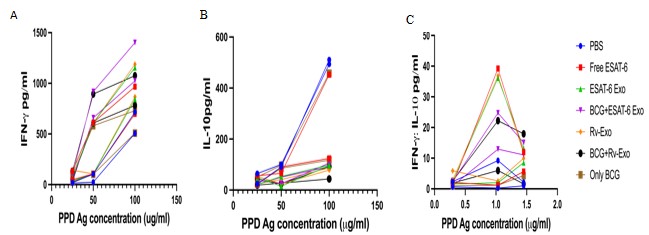

Supplement: Supplementary Figure S3 — The splenocytes of immunized animals of various groups at twelve weeks post infection were stimulated with PPD for 72 hours and splenocytes culture supernatants were collected to assess the IFN-γ (A) and IL-10 (B) cytokines and IFN-γ/IL-10 ratio was also calculated (C). [file Image3.jpeg]

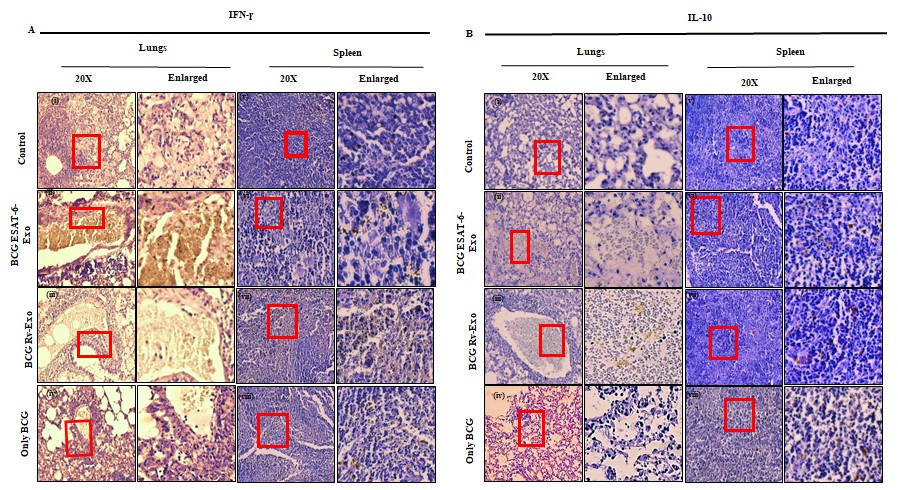

Supplement: Supplementary Figure S4 — The immunohistochemically analysis of lung (A) and spleen (B) tissues from four distinct groups: (A) Control, (B) BCG+ESAT-6 Exo, (C) BCG+Rv-Exo, and (D) Only BCG were assessed using antibodies specific to Th-1 (IFN-γ) and Th-2 (IL-10) cytokines. [file Image4.jpeg]
