# Supplementary material for: Mycobacterium tuberculosis antigen-containing exosomes reinforce BCG vaccine efficacy by augmenting long-term protection and memory response against experimental tuberculosis in BALB-C mice
Source: Front Immunol. 2026 Mar 13;17:1742207. doi: 10.3389/fimmu.2026.1742207 (PMC13021648; doi:10.3389/fimmu.2026.1742207)
Supplement: Supplementary file 8 [file Table1.docx]

| **S. NO** | **Protein ID** | **Name** | **Epitope No** |
| --- | --- | --- | --- |
| 1 | A0A045K7X4 | Putative membrane glycine and proline rich protein OS=Mycobacterium tuberculosis OX=1773 GN=E5M05_10525 PE=4 SV=1 | 6 |
| 2 | A0A0U0R7K6 | Conserved exported protein of uncharacterized function OS=Mycobacterium tuberculosis | 7 |
| 3 | A0A655IS76 | Transporter MMPL8 OS=Mycobacterium tuberculosis | 19 |
| 4 | A0A8E0A753 | Primosome assembly protein PriA OS=Mycobacterium tuberculosis | 14 |
| 5 | A0A062WGY3 | Putative two component sensor kinase OS=Mycobacterium tuberculosis | 7 |
| 6 | A0A045J208 | Deoxyguanosinetriphosphate triphosphohydrolase-like protein OS=Mycobacterium tuberculosis | 15 |
| 7 | A0A8H2FC55 | FAD-binding dehydrogenase (Fragment) OS=Mycobacterium tuberculosis | 17 |
| 8 | R4M8D8 | domain-containing protein OS=Mycobacterium tuberculosis | 6 |
| 9 | A0A109T121 | ATP-dependent helicase OS=Mycobacterium tuberculosis | 48 |
| 10 | R4M9X8 | Pentapeptide repeat-containing protein OS=Mycobacterium tuberculosis | 15 |
| 11 | A0A2I7W4Z3 | D-alanyl-D-alanine-carboxypeptidase/endopeptidase AmpH OS=Mycobacterium tuberculosis | 15 |
| 12 | A0A655JMS4 | Transporter OS=Mycobacterium tuberculosis | 16 |
| 13 | A0A2I7W721 | FeS cluster assembly protein SufB OS=Mycobacterium tuberculosis | 29 |
| 14 | A0A045K6M9 | FdhF/YdeP family oxidoreductase OS=Mycobacterium tuberculosis | 24 |
| 15 | A0A654T4K6 | Acyl-CoA dehydrogenase fadE16 OS=Mycobacterium tuberculosis | 15 |
| 16 | A0A0H3LB85 | . Metal cation transporter P-type ATPase OS=Mycobacterium tuberculosis | 19 |
| 17 | A0A8E0E3Z0 | TfuA domain-containing protein OS=Mycobacterium tuberculosis | 17 |
| 18 | A0A109SUJ0 | Alpha-mannosidase OS=Mycobacterium tuberculosis | 22 |
| 19 | A0A045IRR3 | Acyl-CoA dehydrogenase OS=Mycobacterium tuberculosis | 21 |
| 20 | A0A045HAR6 | 3-methyl-2-oxobutanoate hydroxymethyltransferase OS=Mycobacterium tuberculosis | 7 |
| 21 | A0A109S5T0 | UPF0182 protein RN06_0075 OS=Mycobacterium tuberculosis | 27 |
| 22 | A0A0E8BBK1 | CBS domain-containing protein OS=Mycobacterium tuberculosis | 18 |
| 23 | A0A109STB8 | Fatty-acid--CoA ligase FadD2 OS=Mycobacterium tuberculosis | 11 |
| 24 | A0A045IZM1 | Molybdenum transport system permease OS=Mycobacterium tuberculosis | 10 |
| 26 | R4M831 | Polyketide synthase Pks12 OS=Mycobacterium tuberculosis | 37 |
| 27 | A0A7U8YN47 | Conserved membrane protein OS=Mycobacterium tuberculosis | 35 |
| 28 | A0A0E9AKJ9 | Transporter OS=Mycobacterium tuberculosis | 16 |
| 29 | A0A8E0A588 | Polyketide synthase pks5 OS=Mycobacterium tuberculosis | 37 |
| 30 | A0A0H3L668 | Alpha-mannosidase OS=Mycobacterium tuberculosis | 22 |
| 31 | A0A655HNV3 | Polyketide synthase OS=Mycobacterium tuberculosis | 39 |
| 32 | A0A045H8G1 | Amino acid permease OS=Mycobacterium tuberculosis | 10 |
| 33 | A0A7U8YIN5 | Transmembrane protein OS=Mycobacterium tuberculosis | 5 |
| 34 | A0A0E8X3P7 | .Glutamine--fructose-6-phosphate aminotransferase [isomerizing] OS=Mycobacterium tuberculosis | 12 |
| 35 | A0A2I7W937 | . Phosphate transporter OS=Mycobacterium tuberculosis | 14 |
| 36 | A0A655FSA6 | . Uncharacterized protein OS=Mycobacterium tuberculosis | 6 |
| 37 | A0A045IEJ3 | Chromosome partition protein Smc OS=Mycobacterium tuberculosis | 52 |
| 38 | A0A2I7W7N2 | Polyketide synthase OS=Mycobacterium tuberculosis | 39 |
| 39 | A0A2I7WDZ4 | ESX-2 secretion system protein EccD OS=Mycobacterium tuberculosis | 15 |
| 40 | A0A045J4Z3 | TfuA-like protein OS=Mycobacterium tuberculosis | 20 |
| 41 | A0A0H3LK70 | Uncharacterized protein OS=Mycobacterium tuberculosis | 6 |
| 42 | A0A0H3LBB5 | Sugar-transport integral membrane protein ABC transporter OS=Mycobacterium tuberculosis | 7 |
| 43 | A0A2I7WD95 | Helicase OS=Mycobacterium tuberculosis | 48 |
| 44 | P9WIQ4 | RecBCD enzyme subunit RecC OS=Mycobacterium tuberculosis | 38 |
| 45 | A0A0E8UCW1 | Adenosylcobinamide-GDP ribazoletransferase OS=Mycobacterium tuberculosis | 5 |
| 46 | A0A8E0AGV6 | DeaD/DeaH box family ATP-dependent RNA helicase OS=Mycobacterium tuberculosis | 21 |

**Table-S1: Epitope mapping of H37Rv infected Alveolar macrophage derived exosomal proteins (B cell epitopes)**
