# Supplementary material for: Mycobacterium tuberculosis antigen-containing exosomes reinforce BCG vaccine efficacy by augmenting long-term protection and memory response against experimental tuberculosis in BALB-C mice
Source: Front Immunol. 2026 Mar 13;17:1742207. doi: 10.3389/fimmu.2026.1742207 (PMC13021648; doi:10.3389/fimmu.2026.1742207)
Supplement: Supplementary file 11 [file Table4.docx]

| **Sr. No.** | **Protein Name Absent in BCG** | | **Protein ID** | **References** |
| --- | --- | --- | --- | --- |
| **1.** | **Esx conserved component eccd2. esx-2 type vii secretion system protein. probable transmembrane protein** | | Rv3887c  **A0A1R3Y5J4** | Li, Y., Zeng, J., Shi, J., Wang, M., Rao, M., Xue, C., Du, Y., & He, Z. (2010). A Proteome-Scale Identification of Novel Antigenic Proteins in Mycobacterium tuberculosis toward Diagnostic and Vaccine Development. Journal of Proteome Research, 9(9), 4812–4822. https://doi.org/10.1021/pr1005108 |
| **2.** | **ESX conserved component EccD2 OS=Mycobacterium tuberculosis OX=1773 GN=eccD2 PE=3 SV=1** | | Rv3887c  **A0A045I5U9** | Li, Y., Zeng, J., Shi, J., Wang, M., Rao, M., Xue, C., Du, Y., & He, Z. (2010). A Proteome-Scale Identification of Novel Antigenic Proteins in Mycobacterium tuberculosis toward Diagnostic and Vaccine Development. Journal of Proteome Research, 9(9), 4812–4822. https://doi.org/10.1021/pr1005108 |
| **3.** | **ATPase P OS=Mycobacterium tuberculosis** | | **(**Rv0933)  **A0A045HTY2** | Long, Y., Wang, B., Xie, T., Luo, R., Tang, J., Deng, J., & Wang, C. (2023). Overexpression of efflux pump genes is one of the mechanisms causing drug resistance in Mycobacterium tuberculosis. Microbiology Spectrum, 12(1). https://doi.org/10.1128/spectrum.02510-23 |
| **4.** | **Oxidoreductase OS=Mycobacterium tuberculosis** | | **(** Rv0484c)  **A0A045IQY5** | Long, Y., Wang, B., Xie, T., Luo, R., Tang, J., Deng, J., & Wang, C. (2023). Overexpression of efflux pump genes is one of the mechanisms causing drug resistance in Mycobacterium tuberculosis. Microbiology Spectrum, 12(1). https://doi.org/10.1128/spectrum.02510-23 |
| **5.** | **Cytochrome c biogenesis protein OS=Mycobacterium tuberculosis** | | **(** Rv3673c)  **A0A8D5WZQ2** | Small, J. L., Park, S. W., Kana, B. D., Ioerger, T. R., Sacchettini, J. C., & Ehrt, S. (2013). Perturbation of Cytochrome c Maturation Reveals Adaptability of the Respiratory Chain in Mycobacterium tuberculosis. mBio, 4(5). <https://doi.org/10.1128/mbio.00475-13> |
| **6.** | **Two-component system transcriptional regulator OS=Mycobacterium tuberculosis** | | **(** Rv0981)  **A0A0H3LA31** | Parish, T. (2014). Two-Component regulatory systems of mycobacteria. Microbiology Spectrum, 2(1). <https://doi.org/10.1128/microbiolspec.mgm2-0010-2013> |
| **7.** | **Sugar-transport integral membrane protein ABC transporter OS=Mycobacterium tuberculosis** | | **(**Rv2040c)  **A0A0H3LBB5** | Kim, S., Shin, A., Lee, B., Kim, H., Jeon, B. Y., Cho, S., Park, J., & Shin, S. J. (2009). Characterization of Immune Responses to Mycobacterium tuberculosis Rv2041c Protein. Journal of Bacteriology and Virology, 39(3), 183. <https://doi.org/10.4167/jbv.2009.39.3.183> |
| **8.** | **. Transmembrane protein OS=Mycobacterium tuberculosis** | | **(** Rv0355c)  **A0A2I7WD76** | Sharma, N., Shariq, M., Quadir, N., Singh, J., Sheikh, J. A., Hasnain, S. E., & Ehtesham, N. Z. (2021). Mycobacterium tuberculosis Protein PE6 (Rv0335c), a Novel TLR4 Agonist, Evokes an Inflammatory Response and Modulates the Cell Death Pathways in Macrophages to Enhance Intracellular Survival. Frontiers in Immunology, 12. <https://doi.org/10.3389/fimmu.2021.696491> |
| **9.** | **3-methyl-2-oxobutanoate hydroxylmethyltransferase OS=Mycobacterium tuberculosis** | | **(** Rv0038)  **A0A045HAR6** | Lunge, A., Gupta, R., Choudhary, E., & Agarwal, N. (2020). The unfoldase ClpC1 of Mycobacterium tuberculosis regulates the expression of a distinct subset of proteins having intrinsically disordered termini. Journal of Biological Chemistry, 295(28), 9455–9473. <https://doi.org/10.1074/jbc.ra120.013456> |
| **10.** | | **FAD-binding dehydrogenase (Fragment) OS=Mycobacterium tuberculosis OX=1773 GN=E5M23_16735 PE=4 SV=1** | **(** Rv3129)  **A0A8H2FC55** | Harold, L. K., Antoney, J., Ahmed, F. H., Hards, K., Carr, P. D., Rapson, T., Greening, C., Jackson, C. J., & Cook, G. M. (2018). FAD-sequestering proteins protect mycobacteria against hypoxic and oxidative stress. Journal of Biological Chemistry, 294(8), 2903–5814. <https://doi.org/10.1074/jbc.ra118.006237> |
| **11.** | | **Acyl-CoA dehydrogenase fadE16 OS=Mycobacterium tuberculosis** | **(** Rv1679)  **A0A654T4K6** | Cole, S. T., Brosch, R., Parkhill, J., Garnier, T., Churcher, C., Harris, D., Gordon, S. V., Eiglmeier, K., Gas, S., Barry, C. E., Tekaia, F., Badcock, K., Basham, D., Brown, D., Chillingworth, T., Connor, R., Davies, R., Devlin, K., Feltwell, T., . . . Barrell, B. G. (1998b). Deciphering the biology of Mycobacterium tuberculosis from the complete genome sequence. Nature, 393(6685), 537–544. <https://doi.org/10.1038/31159> |
| **12.** | | **Transmembrane transporter mmpL12 OS=Mycobacterium tuberculosis** | **(** Rv2536)  **A0A7U8U5Q7** | Domenech, P., Reed, M. B., & Barry, C. E. (2005). Contribution of theMycobacterium tuberculosisMMPL protein family to virulence and drug resistance. Infection and Immunity, 73(6), 3492–3501. <https://doi.org/10.1128/iai.73.6.3492-3501.2005> |
